# Evans function and Fredholm determinants

**DOI:** 10.1098/rspa.2014.0597

**Published:** 2015-02-08

**Authors:** Issa Karambal, Simon J. A. Malham

**Affiliations:** Maxwell Institute for Mathematical Sciences, and School of Mathematical and Computer Science, Heriot–Watt University, Edinburgh, UK

**Keywords:** Fredholm determinant, Evans function, travelling waves

## Abstract

We explore the relationship between the Evans function, transmission coefficient and Fredholm determinant for systems of first-order linear differential operators on the real line. The applications we have in mind include linear stability problems associated with travelling wave solutions to nonlinear partial differential equations, for example reaction–diffusion or solitary wave equations. The Evans function and transmission coefficient, which are both finite determinants, are natural tools for both analytic and numerical determination of eigenvalues of such linear operators. However, inverting the eigenvalue problem by the free-state operator generates a natural linear integral eigenvalue problem whose solvability is determined through the corresponding infinite Fredholm determinant. The relationship between all three determinants has received a lot of recent attention. We focus on the case when the underlying Fredholm operator is a trace class perturbation of the identity. Our new results include (i) clarification of the sense in which the Evans function and transmission coefficient are equivalent and (ii) proof of the equivalence of the transmission coefficient and Fredholm determinant, in particular in the case of distinct far fields.

## 1. Introduction

Our goal is to establish the connection between the Evans function, transmission coefficient and Fredholm determinant associated with linear *n*th order eigenvalue problems on R of the form

$$(\partial -A_{0}-V)Y=O.$$

Here, ∂ is the derivative operator ∂*Y* =*Y* ′ and $A_{0}:R\times C\mapsto C^{n\times n}$ and $V:R\mapsto C^{n\times n}$ are bounded multiplicative operators. We suppose that *V* represents a perturbative potential function that decays to zero in the far field of the domain R, while *A*_0_ generates a background or free state. It is constant in the far field though the limits are not necessarily the same. We suppose further that *A*_0_ depends linearly on a spectral parameter λ∈C. Indeed, large classes of eigenvalue problems can be couched in the form above. The problem is to determine those values of λ, eigenvalues, for which suitable integrable solutions $Y\in C^{n}$ exist to the equation above. The Evans function and transmission coefficient are standard tools in this endeavour. Away from the essential spectrum, and suitably scaled, they are analytic functions of the spectral parameter λ whose zeros coincide with eigenvalues. The multiplicity of the zeros coincide with the algebraic multiplicity of the eigenvalues. Modulo a non-zero scalar factor that renders it domain independent, the Evans function is the determinant of the square matrix whose left block is *Y* ^−^ and right block *Y* ^+^. The columns of these two matrices are solutions to the differential equation above that decay to zero exponentially fast in the left and right far fields, respectively. The Evans function measures the ‘distance from intersection’ of the subspaces spanned by the columns of *Y* ^−^ and *Y* ^+^. The transmission coefficient which is also a determinant, measures the degree to which the solutions *Y* ^−^, that decay to zero in the left far field, are orthogonal to the subspace that is orthogonal to the subspace of solutions that decays to zero in the right far field. Unwrapping the two orthogonality conditions explains why the Evans function and transmission coefficient are essentially equivalent. We assume away from the essential spectrum (∂−*A*_0_)^−1^ exists. Then our eigenvalue problem can be expressed in the form (id−(∂−*A*_0_)^−1^*V*)*Y* =*O*, or, with *V* =*U*|*V* | representing the polar decomposition of *V* and setting *ϕ*:=|*V* |^1/2^*Y* , in Birman–Schwinger form

$$(\text{id}-|V{|}^{1/2}{\left(\partial -A_{0}\right)}^{-1}U|V{|}^{1/2})\phi =O.$$

From this perspective, we again seek values of the spectral parameter λ∈C for which solutions to this problem that decay to zero in the far field exist. The natural underlying Hilbert space is $L^{2}(R;C^{n})$. For the applications we have in mind, establishing that |*V* |^1/2^(∂−*A*_0_)^−1^*U*|*V* |^1/2^ is a Hilbert–Schmidt compact operator on this space is relatively straightforward. However, herein we focus on the case when it is a trace class operator, i.e. a nuclear operator. With this property, zeros of the Fredholm determinant of id−|*V* |^1/2^(∂−*A*_0_)^−1^*U*|*V* |^1/2^ coincide with eigenvalues. Thus, we come to the central issue. In what sense are the Evans function, transmission coefficient and Fredholm determinant related? Let us briefly outline what has already been established.

The Evans function was first proposed by Evans [1], while Alexander *et al*. [2] established it as a geometric tool for stability analysis. Subsequently, it has become a standard tool in analytical and numerical studies of the stability of travelling waves; see the review papers featuring the Evans function by Sandstede [3] and Kapitula [4]. The Evans function is also called the miss-distance function [5]. It is also a generalization of the Wronskian and Jost function. The transmission coefficient has it origins much further back in the mathematical literature. Its connection to the Evans function, though trivial in the scalar case, can be found in Swinton [6] and Bridges & Derks [7] for higher order problems. The Fredholm determinant for determining the solvability of linear integral equations was introduced by Fredholm [8]. It has been given recent impetus by Bornemann [9]. Its connection to the transmission coefficient goes back to Jost & Pais [10]. Simon [11,12] computes the explicit relationship between the Fredholm determinant and Wronskian for some example scalar Schrödinger operators; also see Kapitula & Sandstede [13]. However, more generally, Gesztesy & Makarov [14] showed that for operators with semi-separable kernels, their Fredholm and 2-modified Fredholm determinants can be reduced to the determinant of finite rank operators, potentially useful for the evaluation of such Fredholm determinants. Gesztesy *et al.* [15] then established the connection between the Evans function and a 2-modified Fredholm determinant. They also gave a coordinate-free definition of the Evans function as a ratio of the perturbed and unperturbed versions of the function. The 2-modified Fredholm determinant is relevant for their equivalence results as systems of first-order operators generate operators |*V* |^1/2^(∂−*A*_0_)^−1^*U*|*V* |^1/2^ which are Hilbert–Schmidt class and in general not trace class. When such operators are trace class, the Fredholm determinant is the natural object in the equivalence result. Indeed, systems of Schrödinger operators represent an explicit example [16, Section 4].

Our goal herein is to establish a unified picture of the relationship between the Evans function, transmission coefficient and Fredholm determinant. We focus on those systems of first-order operators for which |*V* |^1/2^(∂−*A*_0_)^−1^*U*|*V* |^1/2^ is trace class and the matrix trace of the matrix perturbation potential *V* is zero. By considering this subclass of first-order operators, we gain a degree of clarity and directness. To begin with we assume *A*_0_ is constant, but in our final main §7 we assume distinct far field limits for *A*_0_ which is therefore no longer constant. The free Evans function and free transmission coefficients are the corresponding quantities associated with the operator ∂−*A*_0_. What we achieve in this paper is as follows, we:

(i) Provide practical tests to determine when |*V* |^1/2^(∂−*A*_0_)^−1^*U*|*V* |^1/2^ is trace class. These follow results in Simon [12, ch. 4 (see §3)];
(ii) Show how two important classes of examples, systems of Schrödinger operators and arbitrary order scalar operators, generate operators |*V* |^1/2^(∂−*A*_0_)^−1^*U*|*V* |^1/2^ which are trace class. We reveal how the trace class properties of this Birman–Schwinger formulation naturally reduce to the trace class properties of the example operators directly. These examples also demonstrate how many practical systems generate such trace class operators, with the matrix trace of *V* also equal to zero (see §4);
(iii) Prove simply and directly that the ratio of the Evans function and free Evans function equals the ratio of the transmission coefficient and free transmission coefficient. This new insight clarifies their relationship and indicates a convenient rescaling of the state variables that normalizes the free transmission coefficient to unity (see §5);
(iv) We show the matrix trace of the semi-separable kernels of Birman–Schwinger operators |*V* |^1/2^(∂−*A*_0_)^−1^*U*|*V* |^1/2^ are continuous along the diagonal, despite the fact the kernels have a jump discontinuity there—this assumes the matrix trace of *V* is zero. Hence, we can unambiguously define the trace of such operators. We then provide a simple and direct proof that the scaled transmission coefficient equals the Fredholm determinant of |*V* |^1/2^(∂−*A*_0_)^−1^*U*|*V* |^1/2^, assuming it is trace class (see §6).
(v) Prove, for the case of distinct far fields, the scaled transmission coefficient equals the Fredholm determinant with mild algebraic decay constraints on *V* (see §7).

Items (ii) to (v) above are a self-contained collection of new results. We bookend the sections above with §§2 and 8. In §2, we provide preliminary results characterizing the spaces of trace class and Hilbert–Schmidt class operators and their relation. We include some important inequalities required in subsequent sections. In §8, we summarize our results, discuss conclusions we can draw from them and outline possible future projects.

## 2. Characterizations

To be self-contained, we record a few basic facts on compact operators that we shall need. We refer to Reed & Simon [17,18], Simon [12] and Gohberg *et al.* [19] for more details. Let H denote a separable Hilbert space with unitary basis {*φ*_*m*_}_*m*≥1_ and standard inner product ${\left\langle \cdot ,\cdot \right\rangle }_{H}$. We use $I_{\infty }=I_{\infty }(H)$ to denote the set of compact operators in H. An operator $K\in I_{\infty }$ is positive if ${\left\langle \varphi ,K\varphi \right\rangle }_{H}\geq 0$ for all φ∈H. For any positive operator *K*, there is a unique operator $\sqrt{K}$ such that $K={\left(\sqrt{K}\right)}^{2}$. The adjoint operator *K*^†^ to *K* is the unique operator such that ${\left\langle K^{†}\varphi ,\varphi \right\rangle }_{H}={\left\langle \varphi ,K\varphi \right\rangle }_{H}$ for all φ∈H. The operator *K*^†^*K* is positive as ${\left\langle K^{†}K\varphi ,\varphi \right\rangle }_{H}=∥K\varphi {∥}_{H}^{2}\geq 0$. In particular, we define $|K|=\sqrt{K^{†}K}$. Lastly, there exists a unique unitary operator *U* such that *K*=*U*|*K*|. For any operator $K\in I_{\infty }$, we define its trace by $\mathrm{tr}\;K:=\sum_{m\geq 1}{\left\langle \varphi_{m},K\varphi_{m}\right\rangle }_{H}$. When it exists, the trace is linear and independent of the unitary basis chosen. The Schatten–von Neumann classes of compact operators $I_{p}=I_{p}(H)$ for any *p*≥1 are then defined as follows,

$$I_{p}:=\{K\in I_{\infty }:\mathrm{tr}\;|K{|}^{p}<\infty \}.$$

The set $I_{p}$ equipped with the norm $∥K{∥}_{I_{p}}^{p}:=\mathrm{tr}\;|K{|}^{p}$ is a Banach space. An operator $K\in I_{\infty }$ is *trace class* if it belongs to $I_{1}$ and *Hilbert–Schmidt class* if it belongs to $I_{2}$. The latter class $I_{2}$ is a Hilbert space with inner product ${\left\langle K_{1},K_{2}\right\rangle }_{I_{2}}:=\mathrm{tr}\;K_{1}^{†}K_{2}$. A crucial property of the trace is that the trace of a product composition, of any bounded operator with a trace class operator, is invariant to their permutation. We can also characterize the Schatten–von Neumann classes of compact operators $I_{p}$ as follows. The eigenvalues {λ_*m*_}_*m*≥1_ of any compact operator $K\in I_{\infty }$ are finite in number away from the origin and the origin itself is the only possible accumulation point. The singular values {*s*_*m*_}_*m*≥1_ of $K\in I_{\infty }$ are the eigenvalues of $\sqrt{K^{†}K}$. Then we can equivalently characterize $\mathrm{tr}\;K^{p}=\sum_{m\geq 1}\lambda_{m}^{p}$ and $\mathrm{tr}\;|K{|}^{p}=\sum_{m\geq 1}s_{m}^{p}$. The former is bounded by the latter. There is a natural ordering of the Schatten–von Neumann classes as follows: $I_{p}↪I_{q}$ for any *p*≤*q*. Fundamentally, for any trace class operator $K\in I_{1}$, the Fredholm determinant $\det_{1}(\text{id}+\epsilon K):=\prod_{m\geq 1}(1+\epsilon \lambda_{m})$ is entire in ϵ∈C. Using the relation det exp=exp tr, we can also characterize it (for *p*=1) by

$$\det_{p}(\text{id}+\epsilon K)=\exp\;\sum_{\ell \geq p}\frac{{\left(-1\right)}^{\ell -1}}{\ell }\epsilon^{\ell }\;\mathrm{tr}\;K^{\ell }.$$

When *p* is an integer greater than one, we define the *p*-modified or regularized Fredholm determinants for compact operators $K\in I_{p}$ by this last formula as well, knocking out the lower order non-convergent traces. Three further results will prove very useful to us. First, if $A,B\in I_{2}$, then $AB\in I_{1}$. Second, if A:H→H is a bounded operator and $B\in I_{1}$, then $AB\in I_{1}$ and $BA\in I_{1}$. This is the trace class ideal property. Third, if A:H→H is a bounded operator and $B\in I_{2}$, then $AB\in I_{2}$ and $BA\in I_{2}$. This is the Hilbert–Schmidt ideal property. Indeed, we have

$$∥AB{∥}_{I_{1}}\leq ∥A{∥}_{I_{2}}∥B{∥}_{I_{2}},\;∥AB{∥}_{I_{1}}\leq ∥A{∥}_{\mathrm{op}}∥B{∥}_{I_{1}}\;\text{and}\;∥AB{∥}_{I_{2}}\leq ∥A{∥}_{\mathrm{op}}∥B{∥}_{I_{2}},$$

which also hold for *BA* and where ∥⋅∥_op_ denotes the operator norm. The proof of these three results can be found for example in Conway [20, Section 18].

## 3. Practical tests

The natural setting we require, and which we assume hereafter, is the separable Hilbert space of $C^{n}$-valued square integrable functions $H=L^{2}(R;C^{n})$; see Reed & Simon [18, p. 121] for an example basis. As we will be concerned with kernel functions, we also require the separable Hilbert space $L^{2}(R^{2};C^{n\times n})$ with inner product, for any $G,H\in L^{2}(R^{2};C^{n\times n})$, given by

$${\left\langle G,H\right\rangle }_{L^{2}(R^{2};C^{n\times n})}:=\int_{R^{2}}\mathrm{tr}(G^{†}(x;y)H(x;y))\;dx\;dy.$$

The following fundamental lemma is proved in appendix A.

**Lemma 3.1 (Hilbert–Schmidt class operators)**

*The operator* $K\in I_{\infty }$ *is Hilbert*–*Schmidt if and only if there is a function* $G\in L^{2}(R^{2};C^{n\times n})$ *such that*

$$(K\varphi )(x)=\int_{R}G(x;y)\varphi (y)\;dy,$$

*for all* $\varphi \in L^{2}(R;C^{n})$. *In addition, we have* $∥K{∥}_{I_{2}}=∥G{∥}_{L^{2}(R^{2};C^{n\times n})}$.

The Fourier transform of operators will play a key role in our analysis. We define the Fourier transform $L^{2}(R;C^{n})\to L^{2}(R;C^{n})$ and inverse Fourier transform as the maps $\varphi \mapsto \hat{\varphi }$ and $\hat{\varphi }\mapsto \varphi$, respectively, given by $\hat{\varphi }(\xi ):={\left(2\pi \right)}^{-1/2}\int_{R}\varphi (x)\;e^{-i\xi x}\;dx$ and $\varphi (x):={\left(2\pi \right)}^{-1/2}\int_{R}\hat{\varphi }(\xi )\;e^{ix\xi }\;d\xi$. Suppose an operator $K^{*}:L^{2}(R;C^{n})\to L^{2}(R;C^{n})$ is such that its Fourier transform $\hat{K}=\hat{K}(\xi )$ acts *multiplicatively* in Fourier space, i.e. we have $\hat{K^{*}\varphi }=\hat{K}\hat{\varphi }$, where the product $\hat{K}\hat{\varphi }$ is matrix multiplication. Given any multiplicative operator $\hat{K}=\hat{K}(\xi )$ in Fourier space with integral kernel *G* in physical space, we think of K* as the map taking *φ* to the inverse Fourier transform of $\hat{K}\hat{\varphi }$, or equivalently, K*:*φ*↦(2*π*)^−1/2^G*φ, where G*φ represents the convolution of *G* and *φ*. We note that if $H\in L^{2}(R;C^{n\times n}),$ then

$$((K^{*}H)\varphi )(x)={\left(2\pi \right)}^{-1/2}\int_{R}G(x-y)(H\varphi )(y)\;dy,$$

for all $\varphi \in L^{2}(R;C^{n})$. Hence, the kernel of K*H is (2*π*)^−1/2^*G*(*x*−*y*)*H*(*y*). We now prove the following lemma which is the matrix version of a result given in Simon [12, ch. 4].

**Lemma 3.2 (Practical test for Hilbert–Schmidt class)**

*Suppose* $\hat{K},H\in L^{2}(R;C^{n\times n})$ then $K^{*}H\in I_{2},$ *and indeed we have*

$$∥K^{*}H{∥}_{I_{2}}\leq {\left(2\pi \right)}^{-1/2}∥\hat{K}{∥}_{L^{2}(R;C^{n\times n})}∥H{∥}_{L^{2}(R;C^{n\times n})}.$$

**Proof.**

By direct computation, line by line we successively use the following results: (i) the kernel of K*H is (2*π*)^−1/2^*G*(*x*−*y*)*H*(*y*) and the trace of a product of two operators is invariant to their permutation; (ii) the Cauchy–Bunyakovski–Schwarz inequality in the form tr *A*^†^*B*≤(tr *A*^†^*A*)^1/2^(tr *B*^†^*B*)^1/2^ for any two matrices *A* and *B* [21, p. 289]. We used this inequality with *A*=*G*^†^*G* and *B*=*HH*^†^ and also that tr(*HH*^†^)^†^*HH*^†^≡ tr(*H*^†^*H*)^†^*H*^†^*H*; (iii) The sum of the squares of singular values is less than the square of their sum, i.e. (tr *A*^†^*A*)^1/2^≤tr(*A*^†^*A*)^1/2^; (iv) The Young inequality; (v) That $∥\mathrm{tr}\;G^{†}G{∥}_{L^{1}(R;C)}=∥G{∥}_{L^{2}(R;C^{n\times n})}^{2}$ and (vi) The Plancherel Theorem. The direct computation is as follows:

$$\begin{array}{rl} ∥K^{*}H{∥}_{I_{2}}^{2} & =\frac{1}{2\pi }\int_{R^{2}}\mathrm{tr}((G^{†}G)(x-y)(HH^{†})(y))\;dy\;dx\\ & \leq \frac{1}{2\pi }\int_{R^{2}}{\left(\mathrm{tr}({\left(G^{†}G\right)}^{†}(G^{†}G))(x-y)\right)}^{1/2}{\left(\mathrm{tr}({\left(H^{†}H\right)}^{†}(H^{†}H))(y)\right)}^{1/2}\;dy\;dx\\ & \leq \frac{1}{2\pi }\int_{R^{2}}(\mathrm{tr}\;G^{†}G)(x-y)\cdot (\mathrm{tr}\;H^{†}H)(y)\;dy\;dx\\ & \leq \frac{1}{2\pi }∥\mathrm{tr}\;G^{†}G{∥}_{L^{1}(R;C)}∥\mathrm{tr}\;H^{†}H{∥}_{L^{1}(R;C)}\\ & =\frac{1}{2\pi }∥G{∥}_{L^{2}(R;C^{n\times n})}^{2}∥H{∥}_{L^{2}(R;C^{n\times n})}^{2}\\ & =\frac{1}{2\pi }∥\hat{K}{∥}_{L^{2}(R;C^{n\times n})}^{2}∥H{∥}_{L^{2}(R;C^{n\times n})}^{2}. \end{array}$$

▪

Lemma 3.2 provides us with a test to determine if a given bounded operator is of Hilbert–Schmidt class. In practice, suppose we know the Fourier transform $\hat{K}=\hat{K}(\xi )$ of an operator and we have established it lies in $L^{2}(R;C^{n\times n})$. Further suppose *J*=*J*(*x*) and *H*=*H*(*x*) are bounded multiplicative operators from $L^{2}(R;C^{n})$ to $L^{2}(R;C^{n})$ and $J\in L^{\infty }(R;C^{n\times n})$ and $H\in L^{2}(R;C^{n\times n})$. The kernel of JK*H is (2*π*)^−1/2^*J*(*x*)*G*(*x*−*y*)*H*(*y*). The Hilbert–Schmidt ideal property $∥JK^{*}H{∥}_{I_{2}}\leq ∥J{∥}_{\mathrm{op}}∥K^{*}H{∥}_{I_{2}}$ and lemma 3.2 reveal that

$$∥JK^{*}H{∥}_{I_{2}}\leq {\left(2\pi \right)}^{-1/2}∥J{∥}_{L^{\infty }(R;C^{n\times n})}∥\hat{K}{∥}_{L^{2}(R;C^{n\times n})}∥H{∥}_{L^{2}(R;C^{n\times n})}.$$

We would like an analogous practical test of when an operator such as JK*H from $L^{2}(R;C^{n})$ to $L^{2}(R;C^{n})$ is of trace class. To achieve this, we require the two classical results mentioned above, that the product of two Hilbert–Schmidt class operators is of trace class, and the trace class ideal property. To establish that K*H is trace class, for example where *H*=*H*(*x*) is a bounded multiplicative operator and K* is the operator corresponding to $\hat{K}=\hat{K}(\xi )$ in Fourier space, we naturally require the stronger conditions $\hat{K}\in L_{w}^{2}(R;C^{n\times n})$ and $H\in L_{w}^{2}(R;C^{n\times n})$, i.e. in a weighted square integrable space. More precisely we define

$$L_{w}^{2}(R;C^{n\times n}):=\{F\in L^{2}(R;C^{n\times n}):Fw\in L^{2}(R;C^{n\times n})\},$$

where the weight function *w* is defined via the map *w*:*ξ*↦(1+*ξ*^2^)^1/2^. The practical test takes the following form, which is the matrix version of a result given in Reed & Simon [22, Appendix 2].

**Lemma 3.3 (Practical test for trace class)**

*Suppose* $\hat{K},H\in L_{w}^{2}(R;C^{n\times n})$ *then* $K^{*}H\in I_{1},$ *and there exists a constant* *c*>0 *such that*

$$∥K^{*}H{∥}_{I_{1}}\leq c∥\hat{K}{∥}_{L_{w}^{2}(R;C^{n\times n})}∥H{∥}_{L_{w}^{2}(R;C^{n\times n})}.$$

**Proof.**

We adapt the proof for the scalar case given in Reed & Simon [22, Appendix 2]. Using the practical test for Hilbert–Schmidt class lemma 3.2 and that $L_{w}^{2}(R;C^{n\times n})↪L^{2}(R;C^{n\times n})$, our assumptions on $\hat{K}$ and *H* imply that K*H is of Hilbert–Schmidt class and has integral kernel (2*π*)^−1/2^*G*(*x*−*y*)*H*(*y*). We decompose K*H=*AB* into the product of the two operators *A* and *B* defined as follows, *A*:=K*(1−∂^2^)^1/2^*w*^−1^ and *B*:=*w*(1−∂^2^)^−1/2^*H*, where *w*=*w*(*x*) is the weight function. Then using $∥AB{∥}_{I_{1}}\leq ∥A{∥}_{I_{2}}∥B{∥}_{I_{2}}$ and slightly adapting the proof of lemma 3.2 to take into account that *w*=*w*(*x*) is scalar, we find

$$\begin{array}{rl} ∥K^{*}H{∥}_{I_{1}} & =∥(K^{*}{\left(1-\partial^{2}\right)}^{1/2}w^{-1})(w{\left(1-\partial^{2}\right)}^{-1/2}H){∥}_{I_{1}}\\ & \leq ∥K^{*}{\left(1-\partial^{2}\right)}^{1/2}w^{-1}{∥}_{I_{2}}∥w{\left(1-\partial^{2}\right)}^{-1/2}H{∥}_{I_{2}}\\ & \leq {\left(2\pi \right)}^{-1/2}∥\hat{K}w{∥}_{L^{2}(R;C^{n\times n})}∥w^{-1}{∥}_{L^{2}(R;R)}∥w{\left(1-\partial^{2}\right)}^{-1/2}H{∥}_{I_{2}}\\ & =2^{-1/2}∥\hat{K}{∥}_{L_{w}^{2}(R;C^{n\times n})}∥w{\left(1-\partial^{2}\right)}^{-1/2}H{∥}_{I_{2}}, \end{array}$$

as $∥w^{-1}{∥}_{L^{2}(R;R)}=\pi^{1/2}$. Note in the expression $\hat{K}w$ in the penultimate line, *w*=*w*(*ξ*) represents the Fourier transform of (1−∂^2^)^−1/2^. The action of the operator (1−∂^2^)^−1/2^ is given by convolution with a function $G\in L^{2}(R;R)$. In addition, as its Fourier transform *w*^−1^=*w*^−1^(*ξ*) is analytic in the strip {ξ∈C:|Im(ξ)|<1}, by the Paley–Wiener Theorem [18, Theorem IX.13] we also know that $G\varepsilon \in L^{2}(R;R)$, where ε:x↦exp⁡(|x|/2). The kernel of the operator *w*(1−∂^2^)^−1/2^*H* is (2*π*)^−1/2^*w*(*x*)*G*(*x*−*y*)*H*(*y*) and so

$$∥w{\left(1-\partial^{2}\right)}^{-1/2}H{∥}_{I_{2}}^{2}=\frac{1}{2\pi }\int_{R}\left(\int_{R}(1+x^{2})\;G^{2}(x-y)\;dx\right)(\mathrm{tr}(H^{†}H)(y))\;dy.$$

Using $\int_{R}(1+x^{2})\;G^{2}(x-y)\;dx\leq \int_{R}(1+2x^{2}+2y^{2})G^{2}(x)\;dx\leq c(1+y^{2})$ for some constant *c*>0 as $G,G\varepsilon \in L^{2}(R;R)$, we find $∥w{\left(1-\partial^{2}\right)}^{-1/2}H{∥}_{I_{2}}\leq c∥H{∥}_{L_{w}^{2}(R;C^{n\times n})}$. We now insert this into the estimate above.▪

The trace ideal property of $I_{1}$ implies $∥JK^{*}H{∥}_{I_{1}}\leq ∥J{∥}_{\mathrm{op}}∥K^{*}H{∥}_{I_{1}}$ and thus JK*H is also trace class for any bounded multiplicative operator $J:L^{2}(R;C^{n})\to L^{2}(R;C^{n})$ if K*H is trace class. We end this section with a trace formula and an immediate corollary. A proof is provided in appendix B.

**Lemma 3.4 (Trace formula)**

*Given any integer ℓ*≥2, *suppose that* *K*_1_,*K*_2_,…,*K*_ℓ_ *are Hilbert*–*Schmidt operators with canonical respective kernels* *G*_1_,*G*_2_,…,*G*_ℓ_ in $L^{2}(R^{2};C^{n\times n})$. *Then we have*

$$\mathrm{tr}\;K_{1}K_{2}\cdots K_{\ell }=\mathrm{tr}\;\int_{R^{\ell }}G_{1}(y_{1};y_{2})G_{2}(y_{2};y_{3})\cdots G_{\ell }(y_{\ell };y_{1})\;dy_{\ell }\cdots dy_{1}.$$

*If* *K* *is trace class and its kernel* $G\in C^{n\times n}$ *is continuous on* $R^{2},$ *then* $\mathrm{tr}\;K=\mathrm{tr}\;\int_{R}G(x;x)\;dx$.

**Corollary 3.5 (Trace formula: separable kernel)**

*If a Hilbert–Schmidt operator* *K* *has a separable kernel* *G*(*x*;*y*)=*G*_1_(*x*)*G*_2_(*y*) *for all* $(x,y)\in R^{2},$ *then for any integer* ℓ≥1, *we have*

$$\mathrm{tr}\;K^{\ell }=\mathrm{tr}{\left(\int_{R}G_{2}(y)G_{1}(y)\;dy\right)}^{\ell }.$$

**Remark 3.6**

Some additional comments on the material above are as follows: (i) the map K* above corresponds to the operator *K*(−*i*∇) in Reed & Simon [18, p. 57] and Simon [12, p. 37]; (ii) the sufficiency conditions in lemma 3.3 on $\hat{K}$ and *H* can be weaker, for example a result of Birmann–Solomajk requires they need only be in the space of ℓ^1^-summable $L_{w}^{2}(I_{k};C^{n\times n})$-norms of $\hat{K}$ and *H*, where *I*_*k*_ is the unit interval with centre k∈Z—this space contains $L_{w}^{2}(R;C^{n\times n})$—see Simon [12, ch. 4] for more details. However, the sufficient conditions quoted above are adequate for the applications we have in mind; (iii) the proof of the trace class lemma 3.3 for scalar operators by Reed & Simon [22, Appendix 2] is for any spatial dimension *d*, with weights *w*^*α*^ and *α*>*d*/2; and (iv) in the trace formula lemma 3.4, we relax the continuity condition on *G* in §6 and allow *G* to have a jump across its diagonal—the formula for tr *K* remains meaningful as the kernels we consider have continuous matrix traces along the diagonal; see also remark 6.3 where we discuss results by Brislawn [23,24] for discontinuous kernels.

## 4. Examples

Anticipating our main result in §§6 and 7, we will be concerned with establishing whether operators from $L^{2}(R;C^{n})$ to $L^{2}(R;C^{n})$ of the form |*V* |^1/2^(∂−*A*_0_)^−1^*U*|*V* |^1/2^ are of trace class. Here, $A_{0}\in C^{n\times n}$ is a constant matrix; *V* =*V* (*x*) is a bounded multiplicative matrix operator from $L^{2}(R;C^{n})$ to $L^{2}(R;C^{n})$ and *U*=*U*(*x*) is the unitary matrix obtained from the polar decomposition of *V* . As we saw in §3, the practical tests for Hilbert–Schmidt or trace class operators at our disposal rely on testing the integrability properties of the multiplicative operator (i*ξ*id−*A*_0_)^−1^ in Fourier space corresponding to the operator (∂−*A*_0_)^−1^, as well as the integrability properties of |*V* |^1/2^ and *U*|*V* |^1/2^. We assume in this section that the eigenvalues of *A*_0_ are non-zero and never purely imaginary. Hence, the integrability properties of the operator (i*ξ*id−*A*_0_)^−1^ rely on the rate of its asymptotic decay as |ξ|→∞. We observe (i*ξ*id−*A*_0_)^−1^ is square integrable. We assume *hereafter* that

$$V\in L_{w^{2}}^{1}(R;C^{n\times n})\cap L^{\infty }(R;C^{n\times n})\cap C(R;C^{n\times n}),$$

where $C(R;C^{n\times n})$ represents the space of continuous $C^{n\times n}$-valued matrix functions on R. As $L_{w^{2}}^{1}(R;C^{n\times n})\cap L^{\infty }(R;C^{n\times n})↪L_{w}^{2}(R;C^{n\times n})$ and $L_{w}^{2}(R;C^{n\times n})↪L^{2}(R;C^{n\times n}),$ our functions *V* are square integrable. We observe, using the unitary properties of *U* and that |*V* | is selfadjoint, that $∥U|V{|}^{1/2}{∥}_{L^{2}(R;C^{n\times n})}^{2}=∥|V|{∥}_{L^{1}(R;C^{n\times n})}$. We thus deduce $U|V{|}^{1/2}\in L^{2}(R;C^{n\times n})$ as $L_{w^{2}}^{1}(R;C^{n\times n})↪L^{1}(R;C^{n\times n})$. Further, $|V{|}^{1/2}\in L^{\infty }(R;C^{n\times n})$ by assumption. Hence by the practical test for Hilbert–Schmidt class lemma 3.2, and the immediate discussion after, the operator |*V* |^1/2^(∂−*A*_0_)^−1^*U*|*V* |^1/2^ is of Hilbert–Schmidt class.

Recall the sufficient conditions for |*V* |^1/2^(∂−*A*_0_)^−1^*U*|*V* |^1/2^ to pass the practical test for trace class lemma 3.3. These are first that |*V* |^1/2^ have bounded operator norm, and second that $U|V{|}^{1/2}\in L_{w}^{2}(R;C^{n\times n})$ which is equivalent to $|V{|}^{1/2}\in L_{w}^{2}(R;C^{n\times n})$, which is equivalent to $V\in L_{w^{2}}^{1}(R;C^{n\times n})$. These two conditions are satisfied by our assumptions on the potential function stated above. However, the third condition is not satisfied as ${\left(i\xi \text{id}-A_{0}\right)}^{-1}\notin L_{w}^{2}(R;C^{n\times n})$. Hence in general, without further insight and knowledge, the operator |*V* |^1/2^(∂−*A*_0_)^−1^*U*|*V* |^1/2^ fails the practical test for trace class lemma 3.3. However, we now consider two examples of operators of the form above which pass the practical test for trace class. This is achieved by taking advantage of the structure of (∂−*A*_0_)^−1^ and *V* , as will be apparent.

**Example 4.1 (System of elliptic operators)**

Consider the coupled Schrödinger operator of the form ∂^2^−*d*_0_∂−*c*_0_−*v*, where *c*_0_ and *d*_0_ are constant square matrices and *v* is a matrix potential on R. Such operators, for example, arise in the study of the linear stability of travelling wave solutions to systems of nonlinear reaction–diffusion equations. Linearizing the system of equations about the travelling wave in a co-moving frame and assuming an exponential time dependence with growth λ (the spectral parameter) generates an eigenvalue problem of the form $(\partial^{2}-d_{0}\partial -c_{0}-v)u=\lambda a_{0}^{-1}u$ for the eigenstates *u*. Here, *a*_0_ is the matrix of diffusion coefficients. Replacing $c_{0}+\lambda a_{0}^{-1}\to c_{0}$, the determination of eigenvalues reduces to finding the zeros of the determinant of the operator above. In phase space, the first-order constant coefficient operator corresponding to ∂^2^−*d*_0_∂−*c*_0_ and matrix potential *V* corresponding to *v* have the form

$$\partial -A_{0}=\left(\begin{array}{cc} \partial & -\text{id}\\ -c_{0} & \partial -d_{0} \end{array}\right)\;\text{and}\;V=\left(\begin{array}{cc} O & O\\ v & O \end{array}\right),$$

where *O* represents the zero matrix. The matrices |*V* |^1/2^ and *U* have the form

$$|V{|}^{1/2}=\left(\begin{array}{cc} |v{|}^{1/2} & O\\ O & O \end{array}\right)\;\text{and}\;U=\left(\begin{array}{cc} O & v|v{|}^{-1}\\ v|v{|}^{-1} & O \end{array}\right),$$

which can be verified by direct inspection. Additional direct calculation reveals that

$$|V{|}^{1/2}{\left(\partial -A_{0}\right)}^{-1}U|V{|}^{1/2}=\left(\begin{array}{cc} |v{|}^{1/2}{\left[{\left(\partial -A_{0}\right)}^{-1}\right]}_{1,2}v|v{|}^{-1/2} & O\\ O & O \end{array}\right),$$

where [(∂−*A*_0_)^−1^]_1,2_ represents the top right-hand square matrix block in (∂−*A*_0_)^−1^ whose dimensions are half those of (∂−*A*_0_)^−1^ itself. In Fourier space, (∂−*A*_0_)^−1^ here has the form

$${\left(i\xi \text{id}-A_{0}\right)}^{-1}={\left(\begin{array}{cc} i\xi \text{id} & -\text{id}\\ -c_{0} & i\xi \text{id}-d_{0} \end{array}\right)}^{-1}.$$

Direct computation reveals [(i*ξ*id−*A*_0_)^−1^]_1,2_=((i*ξ*)^2^id−(i*ξ*)*d*_0_−*c*_0_)^−1^. This is the operator in Fourier space corresponding to (∂^2^−*d*_0_∂−*c*_0_)^−1^ as expected. We observe that it is square integrable with respect to the weight *w* as ((i*ξ*)^2^id−(i*ξ*)*d*_0_−*c*_0_)^−1^(1+*ξ*^2^)^1/2^∼−*ξ*^−1^id as ξ→±∞. The trace class property of |*V* |^1/2^(∂−*A*_0_)^−1^*U*|*V* |^1/2^ transfers to the trace class property of |*v*|^1/2^[(∂−*A*_0_)^−1^]_1,2_*v*|*v*|^−1/2^, and the properties we assume for *V* transfer to *v*, and vice versa. Hence by the practical test for trace class lemma 3.3, |*V* |^1/2^(∂−*A*_0_)^−1^*U*|*V* |^1/2^ is of trace class.

**Example 4.2 (High-order scalar operator)**

Consider the scalar *n*th order linear operator given by ∂^*n*^+*a*_*n*−1_∂^*n*−1^+⋯+*a*_1_∂+*a*_0_+*v*, where the *a*_*i*_, *i*=0,…,*n*−1 are scalar constants and *v* is a scalar potential function on R. If we rewrite this as a first-order system in phase space, then the corresponding matrix potential *V* has the same form as that in the last example, with the only non-zero entry being the lower left entry which is *v*; however, this is now the scalar entry in the lower left position and the rest of the *n*×*n* matrix *V* has zero entries. Similarly, the matrices |*V* |^1/2^ and *U* have the same form as in the last example, but the entries shown are scalar with the rest of the matrix entries being zero. Direct calculation reveals that the only non-zero entry in the matrix operator |*V* |^1/2^(∂−*A*_0_)^−1^*U*|*V* |^1/2^ is the top left scalar entry |*v*|^1/2^[(∂−*A*_0_)^−1^]_1,*n*_*v*|*v*|^−1/2^, where [(∂−*A*_0_)^−1^]_1,*n*_ represents the top right-hand scalar entry in the operator (∂−*A*_0_)^−1^. Direct calculation reveals the corresponding entry in the Fourier transform of (∂−*A*_0_)^−1^ is given by [(i*ξ*id−*A*_0_)^−1^]_1,*n*_=((i*ξ*)^*n*^+(i*ξ*)^*n*−1^*a*_*n*−1_+⋯+(i*ξ*)*a*_1_+*a*_0_)^−1^. This is square integrable in Fourier space with respect to the weight *w*. Hence, as in the last example, by the practical test for trace class lemma 3.3, the operator |*V* |^1/2^(∂−*A*_0_)^−1^*U*|*V* |^1/2^ is trace class.

## 5. Evans function and transmission coefficient

Our main practical concern are eigenvalue problems associated with the stability of travelling pulses. A wide class of such eigenvalue problems can be expressed in the following form on R:

$$(\partial -A_{0}-V)Y=O.$$

Here, *A*_0_=*A*_0_(λ) is a constant $C^{n\times n}$-valued matrix which depends linearly on the spectral parameter λ∈C. The $C^{n\times n}$-valued matrix function *V* =*V* (*x*) with x∈R represents a potential perturbation. We assume throughout that *V* is *w*^2^-integrable, uniformly bounded and continuous, exactly as outlined at the beginning of the last examples (§4). A comprehensive reference at this stage for the present material is Sandstede [3]. Our goal is to determine the values of λ for which the operator ∂−*A*_0_−*V* is not invertible, i.e. the spectrum of this operator. The complement to the spectrum in C is the resolvent set. Specifically from the spectrum, we are interested in determining the pure-point spectrum of this operator rather than its complement the essential spectrum. To achieve this, we can construct determinant discriminants which are analytic in the spectral parameter away from the essential spectrum and only zero at pure-point spectrum values. Contour integration using such determinants in the spectral parameter plane then provides a global and local location strategy for the pure-point spectrum. We will assume that away from the essential spectrum, the matrix *A*_0_=*A*_0_(λ) is strictly hyperbolic; this is characteristic of a wide class of travelling pulse stability problems. Hence away from the essential spectrum, the eigenvalue equation above has an exponential dichotomy and the unbounded operator ∂−*A*_0_−*V* is Fredholm with index zero. At pure-point spectrum values, the kernel of ∂−*A*_0_−*V* is non-trivial. The existence of the exponential dichotomy to the eigenvalue equation above, and thus Fredholm property of ∂−*A*_0_−*V* , as well as the locale of the essential spectrum, is determined by the classification of the solutions to the following associated constant coefficient equation which does not exhibit a pure-point spectrum:

$$(\partial -A_{0})Y_{0}=O.$$

The existence of an exponential dichotomy to the eigenvalue equation (with potential *V*) implies that there is a, say, *k*-dimensional subspace of solutions to the eigenvalue equation that decays exponentially as x→−∞, and an (*n*−*k*)-dimensional subspace of solutions that decays exponentially as x→+∞. We denote by *Y* ^−^=*Y* ^−^(*x*;λ) the $C^{n\times k}$-valued function whose column span coincides with the subspace decaying as x→−∞ and by *Y* ^+^=*Y* ^+^(*x*;λ) the $C^{(n-k)\times k}$-valued function whose column span coincides with the subspace decaying as x→+∞. Corresponding subspaces of commensurate dimension exist for the constant coefficient equation, we denote by $Y_{0}^{\pm }=Y_{0}^{\pm }(x;\lambda )$ the corresponding matrix valued functions whose columns span the respective exponentially decaying subspaces. Asymptotically as x→±∞, we have $Y^{\pm }∼Y_{0}^{\pm }$.

From this perspective, the pure-point spectrum is determined by the values of λ∈C for which the two subspaces spanned by the columns of *Y* ^−^ and *Y* ^+^, intersect. In other words, a solution to the eigenvalue problem exists that decays exponentially to zero in both far fields. This is equivalent to the condition that the columns of *Y* ^−^ and *Y* ^+^ are not linearly independent, and a test for that is whether the determinant, of the *n*×*n* matrix whose columns are the columns of *Y* ^−^ and *Y* ^+^, is zero. This intersection property should be *x*-independent and by Liouville’s Theorem an appropriate scalar factor achieves this. This is the Evans function, first introduced by Evans [1]. A comprehensive study is provided in [2].

**Definition 5.1 (Evans function)**

The Evans function is the λ-dependent complex scalar quantity

$$\exp\left(-\mathrm{tr}\;A_{0}(\lambda )x-\int_{0}^{x}\mathrm{tr}\;V(y)\;dy\right)\det(Y^{-}(x;\lambda )\;Y^{+}(x;\lambda ).$$

The free Evans function is that corresponding to the constant coefficient operator ∂−*A*_0_, i.e. corresponding to the free state. If we replace *Y* ^±^ by $Y_{0}^{\pm }$ and set *V* ≡0 in the Evans function, we get the free Evans function defined as $\exp(-\mathrm{tr}\;A_{0}(\lambda )x)\;\det(\begin{array}{cc} Y_{0}^{-}(x;\lambda ) & Y_{0}^{+}(x;\lambda ) \end{array})$.

Associated with the eigenvalue problem above is the adjoint eigenvalue problem given by $(\partial +A_{0}^{†}+V^{†})Z^{†}=O$ or ∂*Z*+*ZA*_0_+*ZV* =*O*, where *Z*^†^ is $C^{n\times 1}$-valued and *Z* is $C^{1\times n}$-valued. The adjoint operator in $L^{2}(R;C^{n})$ to ∂−*A*_0_−*V* is $-\partial -A_{0}^{†}-V^{†}$. We denote by *Z*^−^=*Z*^−^(*x*;λ) and *Z*^+^=*Z*^+^(*x*;λ) the, respectively, $C^{(n-k)\times n}$-valued and $C^{k\times n}$-valued functions whose respective row spans determine solution subspaces to the adjoint eigenvalue problem that decay exponentially as x→−∞ and x→+∞. In addition, we denote by $Z_{0}^{\pm }=Z_{0}^{\pm }(x;\lambda )$ the corresponding matrices for the adjoint constant coefficient equation

$$\partial Z_{0}+Z_{0}A_{0}=O.$$

The solutions $Y_{0}^{\pm }$ and $Z_{0}^{\pm }$ to the constant coefficient problems above satisfy a diagonal relation that will be helpful in our subsequent analysis.

**Lemma 5.2 (Diagonal relation)**

*The solutions* $Y_{0}^{\pm }$ *and* $Z_{0}^{\pm }$ *satisfy the relation*

$$\left(\begin{array}{c} Z_{0}^{+}\\ Z_{0}^{-} \end{array}\right)(Y_{0}^{-}\;Y_{0}^{+})=D,$$

*where* *D* *is a constant diagonal matrix with non-zero entries*.

**Proof.**

Consider any pair of solutions $Y_{0}\in C^{n\times 1}$ and $Z_{0}\in C^{1\times n}$ to their respective constant coefficient problems. Then we see ∂(*Z*_0_ *Y*_0_)=−*Z*_0_*A*_0_*Y*_0_+*Z*_0_*A*_0_*Y*_0_=*O*. Thus, *Z*_0_ *Y*_0_ is constant on R. Each solution *Y*_0_ has the form Uexp⁡(μx), where *μ* is an eigenvalue and $U\in C^{n\times 1}$ a corresponding right eigenvector of *A*_0_. By our strict hyperbolicity assumption, there are *n* independent solutions, and *k* of the eigenvalues have a positive real part. Each solution *Z*_0_, corresponding to an adjoint solution, has the form Wexp⁡(−νx), where *ν* is an eigenvalue and $W\in C^{1\times n}$ a corresponding left eigenvector of *A*_0_. Classically, if *μ*≠*ν* then *WU*=0, while if *μ*=*ν* we have *WU*≠0 [21, p. 405, 523].▪

With all this in hand, we can now motivate and define the transmission coefficient. Starting with the Evans function, we find

$$\begin{array}{rl} & \exp\left(-\mathrm{tr}\;A_{0}(\lambda )x-\int_{0}^{x}\mathrm{tr}\;V(y)\;dy\right)\det(Y^{-}\;Y^{+})\\ & \;=\exp\left(-\mathrm{tr}\;A_{0}(\lambda )x-\int_{0}^{x}\mathrm{tr}\;V(y)\;dy\right)\frac{\det\left((Y_{0}^{-}\;Y_{0}^{+})\left(\begin{array}{c} Z_{0}^{+}\\ Z_{0}^{-} \end{array}\right)\right)}{\det l\left(\left(\begin{array}{c} Z_{0}^{+}\\ Z_{0}^{-} \end{array}\right)(Y_{0}^{-}\;Y_{0}^{+})\right)}\;\det(Y^{-}\;Y^{+})\\ & \;=\exp(-\mathrm{tr}\;A_{0}(\lambda )x)\;\det(Y_{0}^{-}\;Y_{0}^{+})\;\exp\left(-\int_{0}^{x}\mathrm{tr}\;V(y)\;dy\right)\frac{\det\left(\begin{array}{cc} Z_{0}^{+}Y^{-} & Z_{0}^{+}Y^{+}\\ Z_{0}^{-}Y^{-} & Z_{0}^{-}Y^{+} \end{array}\right)}{\det\left(\begin{array}{cc} Z_{0}^{+}Y_{0}^{-} & Z_{0}^{+}Y_{0}^{+}\\ Z_{0}^{-}Y_{0}^{-} & Z_{0}^{-}Y_{0}^{+} \end{array}\right)}. \end{array}$$

Note that the first two factors on the right constitute the free Evans function which is *x*-independent and λ-dependent. We could define a generalized transmission coefficient as the product of the middle exponential term and the numerator in the ratio. The denominator would correspond to a free generalized transmission coefficient. However, classically, we take the limit as x→+∞ in these latter terms. The diagonal relation in lemma 5.2 in particular implies $Z_{0}^{-}Y_{0}^{-}=O$ and $Z_{0}^{+}Y_{0}^{+}=O$, and thus also that $\lim_{x\to +\infty }Z_{0}^{+}Y^{+}=O$. Hence the determinants in the ratio above collapse as x→+∞ (using that the limit and determinant operations commute). Cancelling off the determinant of $Z_{0}^{-}Y_{0}^{+}$ which appears in both the numerator and denominator, we find

$$\begin{array}{rl} & \exp\left(-\mathrm{tr}\;A_{0}(\lambda )x-\int_{0}^{x}\mathrm{tr}\;V(y)\;dy\right)\det(Y^{-}\;Y^{+})\\ & \;\equiv \exp(-\mathrm{tr}\;A_{0}(\lambda )x)\;\det(Y_{0}^{-}\;Y_{0}^{+})\cdot \lim_{x\to +\infty }\exp\left(-\int_{0}^{x}\mathrm{tr}\;V(y)\;dy\right)\frac{\det((Z_{0}^{+}Y^{-})(x;\lambda ))}{\det((Z_{0}^{+}Y_{0}^{-})(\lambda ))}. \end{array}$$

Note by the diagonal relation in lemma 5.2, the quantity $Z_{0}^{+}\;Y_{0}^{-}$ is constant. For completeness, we now define the transmission coefficient and free transmission coefficient.

**Definition 5.3 (Transmission coefficient)**

This is defined as the λ-dependent complex scalar quantity

$$\lim_{x\to +\infty }\exp\left(-\int_{0}^{x}\mathrm{tr}\;V(y)\;dy\right)\det((Z_{0}^{+}Y^{-})(x;\lambda )).$$

The free transmission coefficient is simply the quantity $\det(Z_{0}^{+}Y_{0}^{-}(\lambda ))$.

The relation we derived above establishes that away from the essential spectrum the Evans function can be decomposed as a product of the free Evans function and a ratio of the transmission coefficient and free transmission coefficient. In other words, schematically, we have

$$\frac{\text{Evans function}}{\text{free Evans function}}=\frac{\text{transmission coefficient}}{\text{free transmission coefficient}}.$$

We end this section with three important observations. First, the solutions $Y_{0}^{-}\in C^{n\times k}$, $Y_{0}^{+}\in C^{n\times (n-k)}$, $Z_{0}^{-}\in C^{k\times n}$ and $Z_{0}^{+}\in C^{(n-k)\times n}$ to the constant coefficient problems above have the following explicit special forms. Let $U^{-}\in C^{n\times k}$ and $U^{+}\in C^{n\times (n-k)}$ denote the matrices whose columns are the eigenvectors of *A*_0_, respectively, corresponding to the *k* eigenvalues with positive real part and the *n*−*k* eigenvalues with negative real part. Also let $W^{-}\in C^{n\times k}$ and $W^{+}\in C^{n\times (n-k)}$ denote the matrices whose rows are the left eigenvectors of *A*_0_, respectively, corresponding to the *k* eigenvalues with negative real part and the *n*−*k* eigenvalues with positive real part. Then we have the identifications

$$\left(Y_{0}^{-}\;Y_{0}^{+})\equiv \exp(A_{0}x)(U^{-}\;U^{+})\equiv (U^{-}\exp(\Lambda^{-}x)\;U^{+}\exp(\Lambda^{+}x)\right)$$

and

$$\left(\begin{array}{c} Z_{0}^{+}\\ Z_{0}^{-} \end{array}\right)\equiv \left(\begin{array}{c} W^{+}\\ W^{-} \end{array}\right)\exp(-A_{0}x)\equiv \left(\begin{array}{c} \exp(-\Lambda^{-}x)W^{+}\\ \exp(-\Lambda^{+}x)W^{-} \end{array}\right),$$

where *Λ*^−^ and *Λ*^+^ denote the diagonal matrices of the eigenvalues of *A*_0_ with positive and negative real parts, respectively.

Second, we can rescale the free state solutions $Y_{0}^{\pm }$ and $Z_{0}^{\pm }$ to the constant coefficient problems so they satisfy some unitary relations that are helpful for our subsequent analysis. We choose to rescale the adjoint solutions $Z_{0}^{\pm }$ by rescaling *W*^±^ as follows.

**Definition 5.4 (Unitarily scaled solutions)**

Suppose *D* is the constant diagonal matrix from the diagonal relations lemma 5.2. Let *D*_−_ denote the upper *k*×*k* block of *D* and *D*_+_ the lower (*n*−*k*)×(*n*−*k*) block. We define rescaled solutions ${\hat{W}}^{\pm }$ and correspondingly ${\hat{Z}}_{0}^{\pm }$ by

$${\hat{W}}^{\pm }:=D_{\mp }^{-1}W^{\pm }\;\text{and}\;{\hat{Z}}_{0}^{\pm }:=D_{\mp }^{-1}Z_{0}^{\pm }.$$

Note the solutions ${\hat{Z}}_{0}^{\pm }$ have the same exponential form as $Z_{0}^{\pm }$, but with *W*^±^ replaced by ${\hat{W}}^{\pm }$. The nomination of ${\hat{Z}}_{0}^{\pm }$ as unitarily scaled solutions is justified in the following.

**Lemma 5.5 (Unitary relations)**

*The solutions* $Y_{0}^{\pm }$ *and* ${\hat{Z}}_{0}^{\pm }$ *satisfy the unitary relations*

$$\left(\begin{array}{c} {\hat{Z}}_{0}^{+}\\ {\hat{Z}}_{0}^{-} \end{array}\right)(Y_{0}^{-}\;Y_{0}^{+})=\mathrm{id}\;\text{and}\;(Y_{0}^{-}\;Y_{0}^{+})\left(\begin{array}{c} {\hat{Z}}_{0}^{+}\\ {\hat{Z}}_{0}^{-} \end{array}\right)=\mathrm{id}.$$

*These are generated by corresponding unitary relations satisfied by* *U*^±^ and ${\hat{W}}^{\pm }$.

**Proof.**

From the proof of the diagonal relation lemma 5.2, we already know that

$$\left(\begin{array}{c} W^{+}\\ W^{-} \end{array}\right)(U^{-}\;U^{+})=\left(\begin{array}{cc} D_{-} & O\\ O & D_{+} \end{array}\right).$$

Substituting $W^{\pm }=D_{\mp }{\hat{W}}^{\pm }$ into this relation generates the corresponding unitary relation for ${\hat{W}}^{\pm }$ and *U*^±^, i.e. with the identity matrix on the right. Hence the inverse of the *n*×*n* matrix (*U*^−^ *U*^+^) exists and is given by the corresponding *n*×*n* matrix with ${\hat{W}}^{+}$ and ${\hat{W}}^{-}$ in the upper and lower block positions, respectively, as shown above. The second unitary relation, with the order of the two block matrices with ${\hat{W}}^{\pm }$ and *U*^±^ swapped round, now follows [21, p. 117]. The unitary relations for $Y_{0}^{\pm }$ and ${\hat{Z}}_{0}^{\pm }$ now follow using their explicit exponential forms.▪

Third, in the next section, the unitarily scaled solutions ${\hat{Z}}_{0}^{\pm }$ are the natural choice for constructing the Green’s kernel associated with (∂−*A*_0_)^−1^. They are also natural for establishing the equivalence between the transmission coefficient and corresponding Fredholm determinant, as the corresponding free transmission coefficient is unity with these scaled solutions. Any result we prove using the unitarily scaled solutions we can recover for the original solutions $Z_{0}^{\pm }$ by substituting the relation between the two. This is important, as an edifying feature of the Evans function is that it is analytic in λ away from the essential spectrum and its zeros correspond to eigenvalues of ∂−*A*_0_−*V* with coincident multitude. The solutions $Y_{0}^{\pm }$ and $Z_{0}^{\pm }$ can be chosen to be analytic in λ from the start. The Evans function and free Evans function are independent of the unitary rescaling as they are defined only using *Y* ^±^ and $Y_{0}^{\pm }$, respectively. And, as we should expect, the ratio of the transmission coefficient and free transmission coefficient is also invariant to the unitary rescaling. We return to these points in our Conclusion (§8).

**Remark 5.6**

Classically, the transmission coefficient is defined as follows. Consider the solutions *Y* ^−^∼*Y*^−^_0_ as x→−∞ above. These are Jost solutions. Away from the essential spectrum, asymptotically as x→+∞ we have $Y^{-}∼Y_{0}^{-}a+Y_{0}^{+}b$ where the constant *k*×*k* and (*n*−*k*)×(*n*−*k*) matrices *a*=*a*(λ) and *b*=*b*(λ) are the transmission and reflection matrix coefficients, respectively. For an element of the span of *Y* ^−^ to be an eigenfunction, we require it to be asymptotically in the span of $Y_{0}^{+}b$ or equivalently in the span of $Y_{0}^{+}$, as x→+∞. Or equivalently in this limit, we require an element of the span of *Y* ^−^ to be orthogonal to the subspace of $C^{n}$ that is orthogonal to the subspace spanned by the columns of $Y_{0}^{+}$. In other words, we require an element of the span of *Y* ^−^ to be orthogonal to the subspace spanned by the rows of $Z_{0}^{+}$. The existence of a non-trivial linear combination of columns of *Y* ^−^ to be orthogonal to each row of $Z_{0}^{+}$ amounts to requiring $\det(Z_{0}^{+}Y^{-})$ to be zero in the limit. Modulo the constant non-zero exponential factor, we define the transmission coefficient as this determinant. However we note, using the diagonal relation lemma 5.2 we find $Z_{0}^{+}Y^{-}∼(Z_{0}^{+}Y_{0}^{-})a$. In other words, det(a(λ)) equals the ratio of the transmission coefficient to the free coefficient above. For the classical example of the transmission coefficient for the scalar Schrödinger operator see Kapitula & Sandstede [13], for which tr *V* ≡0. They show in this case, the connection between the Evans function, transmission coefficient and Fredholm determinant (in the following sections). Bridges & Derks [7] show the transmission coefficient equals the Evans function up to a non-zero analytic factor.

## 6. Equivalence theorem

Our goal in this section is to show that the transmission coefficient equals the Fredholm determinant for trace class operators, associated with eigenvalue problems on R of the form

$$(\partial -A_{0}-V)Y=O.$$

The setting is precisely that outlined in §5. Let us now specify which Fredholm determinant we mean. We have already seen that away from the essential spectrum (∂−*A*_0_)^−1^ exists. Hence our eigenvalue problem can be expressed in the form

$$(\text{id}-{\left(\partial -A_{0}\right)}^{-1}V)Y=O.$$

As in §§4 and 5, we assume throughout the potential perturbation *V* is *w*^2^-integrable, uniformly bounded and continuous on R. An equivalent formulation is that of Birman–Schwinger. If we use the polar decomposition for *V* =*U*|*V* | and set *ϕ*:=|*V* |^1/2^*Y* , we see

$$\begin{array}{rl} & \;(\text{id}-{\left(\partial -A_{0}\right)}^{-1}V)Y=O\\ \Leftrightarrow & \;(\text{id}-{\left(\partial -A_{0}\right)}^{-1}U|V{|}^{1/2}|V{|}^{1/2})Y=O\\ \Leftrightarrow & \;(|V{|}^{1/2}-|V{|}^{1/2}{\left(\partial -A_{0}\right)}^{-1}U|V{|}^{1/2}|V{|}^{1/2})Y=O\\ \Leftrightarrow & \;(\text{id}-|V{|}^{1/2}{\left(\partial -A_{0}\right)}^{-1}U|V{|}^{1/2})\phi =O. \end{array}$$

Here, |*V* |^1/2^(∂−*A*_0_)^−1^*U*|*V* |^1/2^ is the Birman–Schwinger operator as considered in §4. To proceed, we establish Green’s kernel corresponding to |*V* |^1/2^(∂−*A*_0_)^−1^*U*|*V* |^1/2^. Suppose (∂−*A*_0_)^−1^ has a representation

$${\left(\partial -A_{0}\right)}^{-1}:\varphi (x)\mapsto \int_{R}G(x;y)\varphi (y)\;dy,$$

for some function $G\in L^{2}(R^{2};C^{n\times n})$, which in this context here without loss of generality, we will also assume is continuously differentiable everywhere except along the diagonal *y*=*x*. Note that *G* also depends on λ through *A*_0_=*A*_0_(λ). However, we suppress the dependence on λ in all the relevant variables for the moment. Classical theory implies that we require *G*=*G*(*x*;*y*) to satisfy the pair of differential equations ∂_*x*_*G*−*A*_0_*G*=*δ*(*x*−*y*)id and −∂_*y*_*G*−*GA*_0_=*δ*(*x*−*y*)id. Two formal calculations that can retrospectively be made rigorous reveal this is the correct prescription for *G*=*G*(*x*;*y*). First, we observe $\left(\partial_{x}-A_{0})\int G(x;y)\varphi (y)\;dy=\int \delta (x-y)\varphi (y)\;dy=\varphi (x\right)$. Second, we observe $\int G(x;y)(\partial_{y}-A_{0})\varphi (y)\;dy=\int (-\partial_{y}G(x;y)-G(x;y)A_{0}))\varphi (y)\;dy=\varphi (x)$. We know the solutions to the equations for Green’s function away from the diagonal already. Indeed, Green’s function *G*=*G*(*x*;*y*) with the correct decay properties in the far field is given by the following semi-separable form which can be confirmed by direct substitution,

$$G(x;y):=\left\{ \begin{array}{ll} -Y_{0}^{-}(x){\hat{Z}}_{0}^{+}(y), & x\leq y,\\ Y_{0}^{+}(x){\hat{Z}}_{0}^{-}(y), & x>y. \end{array}\right.$$

The second relation of the unitary relations in lemma 5.5 is equivalent to $Y_{0}^{-}{\hat{Z}}_{0}^{+}+Y_{0}^{+}{\hat{Z}}_{0}^{-}=\text{id}$. The unitarily scaled solutions ${\hat{Z}}_{0}^{\pm }$ in Green’s kernel thus guarantee the natural jump condition *G*(*x*^+^;*x*)−*G*(*x*^−^;*x*)=id across the diagonal *y*=*x* is satisfied.

**Remark 6.1**

The solutions $Y_{0}^{\pm }$ and ${\hat{Z}}_{0}^{\pm }$ have simple exponential forms as indicated at the end of §5. Indeed using these, we can express *G* in the form *G*=*G*(*x*−*y*), consistent with the kernel result for Hilbert–Schmidt operators stated prior to the practical test for Hilbert–Schmidt class lemma 3.2. Indeed, we note that $Y_{0}^{\pm }(x){\hat{Z}}_{0}^{\mp }(y)\equiv U^{\pm }\exp(\Lambda^{\pm }(x-y)){\hat{W}}^{\mp }$.

Green’s kernel corresponding to |*V* |^1/2^(∂−*A*_0_)^−1^*U*|*V* |^1/2^ has the semi-separable form |*V* (*x*)|^1/2^*G*(*x*;*y*)*U*(*y*)|*V* (*y*)|^1/2^. Upon closer inspection, the unitary relation $Y_{0}^{-}{\hat{Z}}_{0}^{+}+Y_{0}^{+}{\hat{Z}}_{0}^{-}=\text{id}$ implies that the diagonal elements of the Green’s kernel matrix *G*=*G*(*x*;*y*) have a unit jump at the diagonal *y*=*x*, while the off-diagonal elements are continuous. In addition, the unitary relation implies that along the diagonal *y*=*x*, we have

$$\begin{array}{rl} |V(x){|}^{1/2}Y_{0}^{-}{\hat{Z}}_{0}^{+}U(x)|V(x){|}^{1/2}+|V(x){|}^{1/2}Y_{0}^{+}{\hat{Z}}_{0}^{-}U(x)|V(x){|}^{1/2} & =|V(x){|}^{1/2}U(x)|V(x){|}^{1/2}\\ \Rightarrow \;\mathrm{tr}\;|V(x){|}^{1/2}Y_{0}^{-}{\hat{Z}}_{0}^{+}U(x)|V(x){|}^{1/2}+\mathrm{tr}\;|V(x){|}^{1/2}Y_{0}^{+}{\hat{Z}}_{0}^{-}U(x)|V(x){|}^{1/2} & =\mathrm{tr}\;V(x). \end{array}$$

Hence, if assume tr *V* ≡0, then along the diagonal *y*=*x*, we have

$$-\mathrm{tr}\;|V(x){|}^{1/2}Y_{0}^{-}{\hat{Z}}_{0}^{+}U(x)|V(x){|}^{1/2}=\mathrm{tr}\;|V(x){|}^{1/2}Y_{0}^{+}{\hat{Z}}_{0}^{-}U(x)|V(x){|}^{1/2},$$

and thus the matrix trace of the kernel |*V* (*x*)|^1/2^*G*(*x*;*y*)*U*(*y*)|*V* (*y*)|^1/2^ is continuous at the diagonal *y*=*x*. We can thus unambiguously define the trace of |*V* |^1/2^(∂−*A*_0_)^−1^*U*|*V* |^1/2^.

**Definition 6.2 (Trace for discontinuous kernels)**

For potential peturbations *V* which are *w*^2^-integrable, uniformly bounded and continuous on R and with tr *V* ≡0, we define the trace of the linear operator |*V* |^1/2^(∂− *A*_0_)^−1^*U*|*V* |^1/2^, for which (∂−*A*_0_)^−1^ has a kernel with a jump along the diagonal, by

$$\mathrm{tr}(|V{|}^{1/2}{\left(\partial -A_{0}\right)}^{-1}U|V{|}^{1/2}):=\mathrm{tr}\int_{R}(-|V(x){|}^{1/2}Y_{0}^{-}(x){\hat{Z}}_{0}^{+}(x)U(x)|V(x){|}^{1/2})\;dx.$$

**Remark 6.3**

We note the following: (i) consider the examples of trace class operators in §4. Green’s kernels corresponding to |*V* |^1/2^(∂−*A*_0_)^−1^*U*|*V* |^1/2^ are given by |*v*(*x*)|^1/2^*G*_12_(*x*;*y*)*v*(*y*)|*v*(*y*)|^−1/2^ and |*v*(*x*)|^1/2^*G*_1*n*_(*x*;*y*)*v*(*y*)|*v*(*y*)|^−1/2^ in the first and second examples, respectively. They both involve the off-diagonal elements of *G* only and hence these kernels are continuous; (ii) by analogous arguments to those above, if tr *V* ≡0, then along the diagonal *y*=*x* the matrix trace of the kernel *G*(*x*;*y*)*V* (*y*) corresponding to (∂−*A*_0_)^−1^*V* is also continuous. Indeed, using the invariance of the trace to the order of the product of two operators, the matrix traces of *G*(*x*;*y*)*V* (*y*) and |*V* (*x*)|^1/2^*G*(*x*;*y*)*U*(*y*)|*V* (*y*)|^1/2^ are equal along the diagonal *y*=*x*; (iii) consider the scalar Schrödinger operator ∂^2^−*c*_0_−*v* which is a special case of both examples in §4. Re-writing it as a first-order operator and focusing on (∂−*A*_0_)^−1^*V* , we observe if *G*=*G*(*x*−*y*) is the 2×2 Green’s kernel corresponding to (∂−*A*_0_)^−1^, then Green’s kernel corresponding to (∂−*A*_0_)^−1^*V* is the 2×2 matrix whose left column contains *G*_12_*v* and *G*_22_*v* and whose right column is zero. The Fourier transforms of *G*_12_ and *G*_22_ are ${\hat{G}}_{12}(\xi )=-{\left(\xi^{2}-c_{0}\right)}^{-1}$ and ${\hat{G}}_{22}=-i\xi {\left(\xi^{2}-c_{0}\right)}^{-1}$. We note ${\hat{G}}_{12}$ is square integrable with respect to the weight function *w*, but ${\hat{G}}_{12}$ is not. Indeed, they are explicitly given by $G_{12}(x)={\left(\pi /2c_{0}\right)}^{1/2}\exp(-c_{0}^{1/2}|x|)$ and $G_{22}(x)={\left(\pi /2c_{0}\right)}^{1/2}\partial_{x}\exp(-c_{0}^{1/2}|x|)$. From Gohberg *et al.* [19, p. 244], we have the following result for scalar separable integral operators whose kernel functions either side of the diagonal *y*=*x* are continuous, up to and including the diagonal. If the operator is trace class, then the kernel function is continuous on $R^{2}$. Our results in (i) for the corresponding Birman–Schwinger operator are consistent with this. Further, as the scalar operator corresponding to *G*_22_ has a jump along the diagonal *y*=*x*, we conclude it cannot be trace class; and (iv) Brislawn [23,24] has shown how to define the trace of a trace class operator with a kernel that is only square integrable and thus not necessarily continuous. This is achieved by averaging on cubes via the Hardy–Littlewood maximal function. In particular, the plain Volterra operator on $L^{2}([0,1];R)$ with kernel equal to 1 below the diagonal and 0 above, has trace equal to $\frac{1}{2}$. However, it has singular values given by 2/(*π*(2*n*+1)) and thus is not trace class [23, Example 3.2].

We now establish the main result of this section, our equivalence theorem. Before we state and prove it, we require the following key lemma. It concerns the solutions *Y* ^−^ of the eigenvalue problem (∂−*A*_0_−*V*)*Y* =*O* whose column span coincides with the subspace of solutions decaying as x→−∞. Indeed, the crucial insight is that by the variation of constants formula, the solutions *Y* ^−^ satisfy the Volterra integral equation

$$Y^{-}=Y_{0}^{-}+\int_{-\infty }^{x}\exp(A_{0}(x-y))V(y)Y^{-}(y)\;dy.$$

**Lemma 6.4 (Volterra integral equation)**

*Assume* tr *V* ≡0 *and* *A*_0_ *and* *V* =*V* (*x*) *satisfy the conditions stated above. Let* *J* *denote the integral operator with kernel* $H(x-y)|V(x){|}^{1/2}\exp(A_{0}(x-y))U(y)|V(y){|}^{1/2},$ *where* *H*=*H*(*x*) *is the Heaviside step function. First, if we set* *ϕ*^−^:=|*V* |^1/2^*Y* ^−^ *and* *ϕ*^−^_0_:=|*V* |^1/2^*Y*^−^_0_, *then* *Y* ^−^ *solves the eigenvalue problem* (*∂*− *A*_0_−*V*)*Y* =*O* *with* *Y* ^−^∼*Y*^−^_0_ *as* x→−∞ *if and only if* *ϕ*^−^ *satisfies*

$$\phi^{-}=\phi_{0}^{-}+J\phi^{-}.$$

*Equivalently we have* $\phi_{0}^{-}=(\mathrm{id}-J)\phi^{-}$. *Second, an equivalent expression for the kernel for* *J* *is given by* $H(x-y)(\phi_{0}^{-}(x){\hat{Z}}_{0}^{+}(y)+\phi_{0}^{+}(x){\hat{Z}}_{0}^{-}(y))U(y)|V(y){|}^{1/2}$, *where* *ϕ*^+^_0_:=|*V* |^1/2^*Y*^+^_0_.

**Proof.**

Preceding the lemma, we already established that *Y* ^−^ satisfies the Volterra integral equation shown if and only if *Y* ^−^ solves the eigenvalue problem (∂−*A*_0_− *V*)*Y* =*O* with *Y* ^−^∼*Y*^−^_0_ as x→−∞. To show *Y* ^−^ satisfies the Volterra integral equation preceding the lemma if and only if *ϕ*^−^ satisfies the Volterra integral equation shown in the lemma, follows by premultiplying the Volterra integral equation for *Y* ^−^ by |*V* (*x*)|^1/2^ and using the definition of *ϕ*^−^. The equivalence of the kernels follows from the unitary relation $U^{-}{\hat{W}}^{+}+U^{+}{\hat{W}}^{-}=\text{id}$ and that $Y_{0}^{\pm }(x)=\exp(A_{0}x)U^{\pm }$ and ${\hat{Z}}_{0}^{\pm }(y)={\hat{W}}^{\pm }\exp(-A_{0}y)$.▪

**Theorem 6.5 (Equivalence)**

*Assume the operator |V |*^1/2^(*∂−A*_0_)^−1^*U|V |*^1/2^ *is trace class, tr V ≡0 and A*_0_ *and V =V (x) satisfy the conditions stated above. Then the transmission coefficient for the eigenvalue problem (∂−A*_0_*−V)Y =O and Fredholm determinant of id−|V |*^1/2^(*∂−A*_0_)^−1^*U|V |*^1/2^ *are equal:
*

$$\lim_{x\to +\infty }\det(({\hat{Z}}_{0}^{+}Y^{-})(x))=\det_{1}(\mathrm{id}-|V{|}^{1/2}{\left(\partial -A_{0}\right)}^{-1}U|V{|}^{1/2}).$$

**Proof.**

First, we focus on the transmission coefficient. As we have ∂*Y* ^−^=(*A*_0_+ *V*)*Y* ^−^ and $\partial {\hat{Z}}_{0}^{+}=-{\hat{Z}}_{0}^{+}A_{0}$, the product rule implies $\partial ({\hat{Z}}_{0}^{+}Y^{-})={\hat{Z}}_{0}^{+}VY^{-}$. Using the unitary relations in lemma 5.5, we know $\lim_{x\to -\infty }({\hat{Z}}_{0}^{+}Y^{-})(x)=\text{id}$ and hence

$$\lim_{x\to +\infty }\;\det(({\hat{Z}}_{0}^{+}Y^{-})(x))=\det\left(\text{id}+\int_{R}({\hat{Z}}_{0}^{+}VY^{-})(y)\;dy\right).$$

We can also establish this result by premultiplying the integral equation for *Y* ^−^ by ${\hat{Z}}_{0}^{+}$, using the unitary relations, and then applying the commutable operations of large *x* limit and determinant.

Second, we focus on the Fredholm determinant. The key observation is that we can decompose the operator |*V* |^1/2^(∂−*A*_0_)^−1^*U*|*V* |^1/2^ as follows. Direct computation using the kernel |*V* (*x*)|^1/2^*G*(*x*;*y*)*U*(*y*)|*V* (*y*)|^1/2^, where *G* is the kernel corresponding to (∂−*A*_0_)^−1^, reveals

$$|V{|}^{1/2}{\left(\partial -A_{0}\right)}^{-1}U|V{|}^{1/2}=J-R,$$

where *J* is the Volterra integral operator given in the Volterra integral equation lemma 6.4 and *R* is the integral operator with kernel given by

$$\phi_{0}^{-}(x){\hat{Z}}_{0}^{+}(y)U(y)|V(y){|}^{1/2}.$$

Note *R* has separable kernel and is thus a finite rank operator and trace class. As we have $∥J{∥}_{I_{1}}\leq ∥|V{|}^{1/2}{\left(\partial -A_{0}\right)}^{-1}U|V{|}^{1/2}{∥}_{I_{1}}+∥R{∥}_{I_{1}}$ and |*V* |^1/2^(∂−*A*_0_)^−1^*U*|*V* |^1/2^ is trace class by assumption, we deduce *J* is also trace class. Using the product decomposition id−*J*+*R*=(id−*J*)(id+(id−*J*)^−1^*R* and provided *J* and (id−*J*)^−1^*R*) are trace class, we have

$$\begin{array}{rl} \det_{1}(\text{id}-J+R) & =\det_{1}((\text{id}-J)(\text{id}+{\left(\text{id}-J\right)}^{-1}R))\\ & =\det_{1}(\text{id}-J)\;\det_{1}(\text{id}+{\left(\text{id}-J\right)}^{-1}R). \end{array}$$

As we see presently (id−*J*)^−1^*R* is trace class as it has a separable kernel.

Third, we compute det_1_(id−*J*). Using the relation det exp=exp tr, we have

$$\log\;\det_{1}(\text{id}-J)=-\sum_{\ell \geq 1}\frac{1}{\ell }\;\mathrm{tr}\;J^{\ell }.$$

As *J* is a Volterra integral operator involving the Heaviside function, using the trace power formula lemma 3.4, we observe that tr *J*^ℓ^ will involve an integral over $R^{\ell }$ of an integrand with a factor *H*(*y*_1_−*y*_2_)*H*(*y*_2_−*y*_3_)⋯*H*(*y*_ℓ−1_−*y*_ℓ_)*H*(*y*_ℓ_−1) of a product of Heaviside functions. Hence for all ℓ≥2, the quantity tr *J*^ℓ^ is zero as it is an integral of an integrand which is zero everywhere except on a subset of ℓ-dimensional measure zero. We also observe tr *J*=0. This follows using the unitary relation in lemma 5.5 and the assumption tr *V* ≡0 which imply the matrix trace of the kernel of *J* is continuous on the diagonal. Hence, we deduce det_1_(id−*J*)=1.

Fourth, we establish that (id−*J*)^−1^*R* is trace class and compute det_1_(id+(id−*J*)^−1^*R*). Using that $\phi_{0}^{-}=(\mathrm{id}-J)\phi^{-}$ from the Volterra integral equation lemma 6.4, we see

$$\begin{array}{rl} \left({\left(\text{id}-J\right)}^{-1}(R\varphi ))(x\right) & ={\left(\text{id}-J\right)}^{-1}\phi_{0}^{-}(x)\int_{R}{\hat{Z}}_{0}^{+}(y)U(y)|V(y){|}^{1/2}\varphi (y)\;dy\\ & =\phi^{-}(x)\int_{R}{\hat{Z}}_{0}^{+}(y)U(y)|V(y){|}^{1/2}\varphi (y)\;dy. \end{array}$$

Hence, (id−*J*)^−1^*R* has separable kernel $\phi^{-}(x){\hat{Z}}_{0}^{+}(y)U(y)|V(y){|}^{1/2}$ and is thus trace class. Then, using the result for separable kernels in corollary 3.5, we find for any *ε*>0 sufficiently small,

$$\begin{array}{rl} \log\;\det_{1}(\text{id}+\epsilon {\left(\text{id}-J\right)}^{-1}R) & =\sum_{\ell \geq 1}\frac{{\left(-1\right)}^{\ell -1}}{\ell }\epsilon^{\ell }\;\mathrm{tr}{\left({\left(\text{id}-J\right)}^{-1}R\right)}^{\ell }\\ & =\sum_{\ell \geq 1}\frac{{\left(-1\right)}^{\ell -1}}{\ell }\epsilon^{\ell }\;\mathrm{tr}{\left(\int_{R}{\hat{Z}}_{0}^{+}(y)U(y)|V(y){|}^{1/2}\phi^{-}(y)\;dy\right)}^{\ell }\\ & =\log\;\det\left(\text{id}+\epsilon \int_{R}({\hat{Z}}_{0}^{+}VY^{-})(y)\;dy\right). \end{array}$$

By analytic continuation, we can extend this result to *ε*=1. Then removing the logarithms, this equals our expression above for the transmission coefficient. ▪

**Remark 6.6**

We have the following observations on the results above: (i) at the core of the equivalence theorem is, for solutions decaying in the far field, on one hand we have the Fredholm determinant associated with the solvability of (id−*J*+*R*)*ϕ*=*O*, while on the other we have the transmission coefficient associated with the solvability of $(\text{id}-J)\phi^{-}=\phi_{0}^{-}$; (ii) we note we have (∂−*A*_0_)|*V* |^−1/2^*J*|*V* |^1/2^=*V* and (∂−*A*_0_)|*V* |^−1/2^*R*|*V* |^1/2^=*O*; (iii) in essence, in the final calculation in the proof of theorem 6.5, we demonstrate that the trace of any power of (id−*J*)^−1^*R* equals the trace of the corresponding power of $\int ({\hat{Z}}_{0}^{+}VY^{-})(y)\;dy$. Hence, we could prove the equivalence of the determinants using the Plemelj–Smithies formula for det_1_(id+*ε*(id−*J*)^−1^*R*) which is analytic in C [25, Theorem 6.8]; (iv) as we can swap the order of two arguments under the trace, using the trace formula lemma 3.4, we observe the traces of all powers of |*V* |^1/2^(∂−*A*_0_)^−1^*U*|*V* |^1/2^ and (∂−*A*_0_)^−1^*V* coincide. Hence, the Fredholm determinants of the two operators coincide, assuming the Fredholm determinant of id−(∂−*A*_0_)^−1^*V* exists; (v) if tr *V* ≠0, then we need to include the factor exp⁡(−tr J) in the evaluation of the Fredholm determinant; (vi) the approach we used in the proof of the equivalence theorem is based on the standard approach—decomposing the given operator with semi-separable kernel into the sum of a Volterra and finite rank operators—which is given in Gesztesy *et al.* [15] for Hilbert–Schmidt operators. They use results from Gesztesy & Makarov [14], Gohberg *et al.* [26] and Gohberg *et al.* [19]; and (vii) Gesztesy *et al.* [15] do not assume strict hyperbolicity which we have done to keep the arguments as succinct as possible.

## 7. Distinct far fields

Our goal in this section is to show that the equivalence of the transmission coefficient and Fredholm determinant carries over to the case when the far field limits of the eigenvalue problem (∂−*A*_0_−*V*)*Y* =*O* are distinct. The Evans function and transmission coefficient are well defined in this instance with only minor modification to their construction in §5—which is the standard approach. What underlies our approach here is that we decompose *A*_0_+*V* in such a way that *A*_0_=*A*_0_(*x*) ensures the distinct far field limits are satisfied so *V* →*O* as x→±∞. We assume this decomposition in this section, as it also gives us the natural framework to show the equivalence of the transmission coefficient and a Fredholm determinant with only a slight modification to the arguments in §6. We also assume in each far field, *A*_0_ is constant and strictly hyperbolic, with the same hyperbolic splitting (the number of eigenvalues with positive real parts, say *k*, and negative real parts, consequently *n*−*k*). We can thus also assume *V* →*O* as x→±∞. Further, we assume both *A*_0_ and *V* are continuous and uniformly bounded. To construct the Evans function or transmission coefficient, we do not need to perform this decomposition; however, the properties we derive for the solutions of the equations

$$\partial Y_{0}=A_{0}Y_{0}\;\text{and}\;\partial Z_{0}=-Z_{0}A_{0}$$

are crucial to our equivalence proof. We now have two separate constant coefficient equations in the far field corresponding to ∂*Y*_0_=*A*_0_*Y*_0_. However as before in §5, given the identical hyperbolic splitting of the two far field limits of *A*_0_, there is *k*-dimensional subspace of solutions that decays exponentially as x→−∞, and an (*n*−*k*)-dimensional subspace of solutions that decays exponentially as x→+∞. We suppose these subspaces are given by the column span of solutions $Y_{0}^{-}\in C^{n\times k}$ and $Y_{0}^{+}\in C^{n\times (n-k)}$, respectively. For the adjoint equation ∂*Z*_0_=−*Z*_0_*A*_0_, there exist solutions collected as rows in the matrices $Z_{0}^{-}\in C^{(n-k)\times n}$ and $Z_{0}^{+}\in C^{k\times n}$ which decay as x→−∞ and x→+∞, respectively. Solutions of commensurate dimension *Y* ^±^ exist to the full problem (∂−*A*_0_−*V*)*Y* =*O* such that $Y^{\pm }∼Y_{0}^{\pm }$ as x→±∞.

We can recover the unitary relations of lemma 5.5 for $Y_{0}^{\pm }$ and suitably defined scaled solutions ${\hat{Z}}_{0}^{\pm }$ despite distinct far fields. The diagonal relation for $Y_{0}^{\pm }$ and $Z_{0}^{\pm }$ becomes the following.

**Lemma 7.1 (Diagonal block relation)**

*The solutions* $Y_{0}^{\pm }$ *and* $Z_{0}^{\pm }$ *satisfy the relation*

$$\left(\begin{array}{c} Z_{0}^{+}\\ Z_{0}^{-} \end{array}\right)(Y_{0}^{-}\;Y_{0}^{+})=\left(\begin{array}{cc} D_{-} & O\\ O & D_{+} \end{array}\right),$$

*where* *D*_±_ *are constant matrices*.

**Proof.**

We observe, by direct computation for any pair of solutions $Y_{0}\in C^{n\times 1}$ and $Z_{0}\in C^{1\times n}$, that ∂(*Z*_0_*Y*_0_)=*O* and so *Z*_0_*Y*_0_ is constant. Asymptotically, we have $Y_{0}^{-}∼\exp(A_{0}(-\infty )x)U^{-}$ and $Z_{0}^{-}∼W^{-}\exp(-A_{0}(-\infty )x)$ where the columns of *U*^−^ and rows of *W*^−^ are the right and left eigenvectors of $A_{0}(-\infty )$. Similarly, we have $Y_{0}^{+}∼\exp(A_{0}(+\infty )x)U^{+}$ and $Z_{0}^{+}∼W^{+}\exp(-A_{0}(+\infty )x)$ where the columns of *U*^+^ and rows of *W*^+^ are the right and left eigenvectors of $A_{0}(+\infty )$. Then using that *W*^−^*U*^−^=*O* and *W*^+^*U*^+^=*O*, we conclude $Z_{0}^{-}Y_{0}^{-}=O$ and $Z_{0}^{+}Y_{0}^{+}=O$. The remaining constant matrices are thus $Z_{0}^{+}Y_{0}^{-}=D_{-}$ and $Z_{0}^{-}Y_{0}^{+}=D_{+}$.▪

Note that the constant matrices *D*_±_ are in general no longer diagonal as $Y_{0}^{-}$ and $Z_{0}^{-}$, and, $Y_{0}^{+}$ and $Z_{0}^{+}$ satisfy different asymptotic problems. We must keep in mind that $Y_{0}^{\pm }$ and $Z_{0}^{\pm }$ are the solutions generated by *A*_0_=*A*_0_(*x*). We *assume* the constant matrices $Z_{0}^{+}Y_{0}^{-}=D_{-}$ and $Z_{0}^{-}Y_{0}^{+}=D_{+}$ are *non-singular*. Generically, this will be the case—see the discussion in §8. This guarantees the free transmission coefficient is non-zero. Using the diagonal block relation lemma 7.1, it guarantees the free Evans function is non-zero. This guarantees (∂−*A*_0_)^−1^ exists (this was automatic in the equal far field case under the strict hyperbolicity assumption on the constant matrix *A*_0_). With this proviso, the block diagonal relation implies the framework we provided in §5 for establishing the relation between the Evans function and the transmission coefficient carries through essentially unchanged. We can mirror all the results in §5 up to and including the schematic formula between the Evans function and transmission coefficient. Then again with the same proviso, the scaled unitary solutions are defined as for the equal far field case in §5 by ${\hat{W}}^{\pm }:=D_{\mp }^{-1}W^{\pm }$ and ${\hat{Z}}_{0}^{\pm }:=D_{\mp }^{-1}Z_{0}^{\pm }$.

**Lemma 7.2 (Unitary relations reprise)**

*Unitary relations for* $Y_{0}^{\pm }$ *and* ${\hat{Z}}_{0}^{\pm }$ *hold for distinct far fields*.

**Proof.**

Making the corresponding change of variables in the diagonal block relation above generates the same diagonal block relation but with ${\hat{Z}}_{0}^{\pm }$ in place of $Z_{0}^{\pm }$ and the appropriate identity matrices in place of *D*_±_ on the right. The two matrices on the left of the new diagonal block relation are then inverses of each other and the unitary relation in reverse order follows.▪

The framework we provided in §6 for establishing the relation between the transmission coefficient and the Fredholm determinant det_1_(id−|*V* |^1/2^(∂−*A*_0_)^−1^*U*|*V* |^1/2^), where now *A*_0_=*A*_0_(*x*) carries through with only a couple of modifications which we now outline. Green’s function *G*=*G*(*x*,*y*) associated with (∂−*A*_0_)^−1^ has the same semi-separable form except that $Y_{0}^{\pm }$ and ${\hat{Z}}_{0}^{\pm }$ are now the solutions generated by *A*_0_=*A*_0_(*x*). Again the unitary relations imply the jump condition for *G* across the diagonal line *y*=*x* is satisfied. If we assume tr *V* ≡0, then the trace of |*V* |^1/2^(∂−*A*_0_)^−1^*U*|*V* |^1/2^ or (∂−*A*_0_)^−1^*V* is defined in the same way. The Volterra integral equation $\phi^{-}=\phi_{0}^{-}+J\phi^{-}$ of lemma 6.4 still applies but now only for *J* defined via the kernel $H(x-y)(\phi_{0}^{-}(x)Z_{0}^{+}(y)+\phi_{0}^{+}(x)Z_{0}^{-}(y))U(y)|V(y){|}^{1/2}$. This requires independent proof which does not rely on *A*_0_ being constant as follows. Direct computation using the unitary relations and that $(\partial -A_{0})Y_{0}^{\pm }=O$ reveals

$$\begin{array}{rl} & \left(\partial -A_{0})(Y_{0}^{-}+|V{|}^{-1/2}J|V{|}^{1/2}Y^{-})(x\right)\\ & \;=(\partial -A_{0})(|V{|}^{-1/2}J|V{|}^{1/2}Y^{-})(x)\\ & \;=(\partial -A_{0})\int_{-\infty }^{x}(Y_{0}^{-}(x){\hat{Z}}_{0}^{+}(y)+Y_{0}^{+}(x){\hat{Z}}_{0}^{-}(y))V(y)Y^{-}(y)\;dy\\ & \;=(Y_{0}^{-}(x){\hat{Z}}_{0}^{+}(x)+Y_{0}^{+}(x){\hat{Z}}_{0}^{-}(x))V(x)Y^{-}(x)\\ & \;=V(x)Y^{-}(x). \end{array}$$

Hence, if $Y^{-}=Y_{0}^{-}+|V{|}^{-1/2}J|V{|}^{1/2}Y^{-}$, we have just shown that (∂−*A*_0_)*Y* ^−^=*V* *Y* ^−^. Premulti-plying this Volterra equation for *Y* ^−^ by |*V* |^1/2^ and using the definitions for *ϕ*^−^ and *ϕ*^−^_0_ gives the result. The proof of the equivalence theorem 6.5 now follows step by step with the solutions $Y_{0}^{\pm }$ and ${\hat{Z}}_{0}^{\pm }$ those generated by *A*_0_=*A*_0_(*x*). We summarize these conclusions as follows.

**Theorem 7.3 (Equivalence reprised)**

*Assume ∂−A*_0_ *is invertible, |V |*^1/2^(*∂−A*_0_)^−1^*U|V |*^1/2^ *is trace class, tr V ≡ 0, A*_0_*=A*_0_(*x) and V =V (x) are both continuous and uniformly bounded and V =V (x) is w*^2^*-integrable. Further assume in each far field A*_0_ *is constant and strictly hyperbolic, with the same hyperbolic splitting, and V →O as* x→±∞*. Then the transmission coefficient for the eigenvalue problem (∂−A*_0_*−V)Y =O and Fredholm determinant of id−|V |*^1/2^(*∂−A*_0_)^−1^*U|V |*^1/2^ *are equal.*

## 8. Conclusion

We have established the equivalence of the Evans function and transmission coefficient for the eigenvalue problem (∂−*A*_0_−*V*)*Y* =*O*, in the sense that the ratio of the Evans function to the free Evans function is equal to the ratio of the transmission coefficient to the free transmission coefficient. As we remarked at the end of §5, the Evans function and free Evans function are invariant to the unitary rescaling, as is the ratio of the transmission coefficient to the free transmission coefficient. Hence, the Fredholm determinant det_1_(id−|*V* |^1/2^(∂−*A*_0_)^−1^*U*|*V* |^1/2^), which equals the transmission coefficient with unitarily scaled solutions for which the free transmission coefficient is unity, equals the ratio of the transmission coefficient to the free transmission coefficient—whether the unitary scaling is used or not. These statements hold for equal or distinct far fields. In other words, we have established that

$$\frac{\text{Evans function}}{\text{Free Evans function}}=\frac{\text{Transmission coefficient}}{\text{Free transmission coefficient}}=\text{Fredholm determinant}.$$

Let us now pull back our perspective to our original problem of determining values of λ∈C for which there exist solutions to (∂−*A*_0_−*V*)*Y* =*O* where *A*_0_=*A*_0_(*x*;λ) in general. The locale of the essential spectrum is determined by the values of λ for which the far field limits of *A*_0_ are no longer strictly hyperbolic—at least one eigenvalue of either limit becomes pure imaginary characterizing the continuous spectrum or the hyperbolic splitting no longer matches. Away from the essential spectrum, we must choose *A*_0_=*A*_0_(*x*;λ) suitably so that (∂−*A*_0_)^−1^ exists—as mentioned previously this is not an issue in the equal far field case. Hence, away from the essential spectrum, the free Evans function and free transmission coefficients are bounded and non-zero. As the Evans function is analytic in that region, the product of the free Evans function and the ratio of the transmission and free transmission coefficients is analytic there, as well as the product of the free Evans function and the Fredholm determinant. Zeros of these product quantities must thus coincide in this region. Those zeros coincide with pure point eigenvalues with coincident multiplicity. We remark that for eigenvalue problems of the form (∂−*A*_0_−*V*)*Y* =*O* that arise in the study of the stability of travelling waves, the origin is an eigenvalue associated with translation invariance. In some cases, the origin is embedded in the essential spectrum within which further analysis of all the discriminants above is required. Some instructive explicit examples illustrating the relation, between the Evans function and transmission coefficient can be found in Kapitula & Sandstede [13] and Kapitula [4], between the transmission coefficient and Fredholm determinant can be found in Simon [12, p. 51] and between the Evans function and 2-modified Fredholm determinant in Gesztesy *et al.* [15] and Gesztesy *et al.* [16].

For the eigenvalue problem (∂−*A*_0_−*V*)*Y* =*O* where *A*_0_=*A*_0_(*x*;λ) and λ∈C is the spectral parameter, our interest surrounds families of operators ∂−*A*_0_−*V* parametrized by λ. For example, the analyticity of the Evans function means we can conduct a global search for eigenvalues via contour integration and the residue theorem in any subregion away from the essential spectrum. Here, the family of operators consists of those associated with values of λ parametrizing the boundary contour of the subregion. Hence the object of interest is a determinant line bundle. See Quillen [27], Jost [28, Section 6.9], Deng & Nii [29] and Alexander *et al.* [2] for constructions of such line bundles. In Alexander *et al.*’s line bundle construction, the fibres are explicitly the Evans function. These constructions suggest we define, away from the essential spectrum, the determinant of the unbounded Fredholm operator ∂−*A*_0_−*V* by the Evans function corresponding to the problem (∂−*A*_0_−*V*)*Y* =*O*, i.e. we set

$$\det(\partial -A_{0}-V):=\text{Evans function},$$

or for a coordinate-free definition, as the ratio of the Evans function to the free Evans function. With the definition above, the determinant $\det(\partial -A_{0})$ is the free Evans function. And in this paper, assuming tr *V* ≡0, we have established that

$$\det(\partial -A_{0}-V)=\det(\partial -A_{0})\cdot \det_{1}(\text{id}-|V{|}^{1/2}{\left(\partial -A_{0}\right)}^{-1}U|V{|}^{1/2},$$

representing the natural determinant multiplicative property for such operators. Elliptic operators for which this does not hold, i.e. when there are multiplicative anomalies, is a research field in itself with important applications in mathematical physics. Here, the zeta-regularized, Quillen, Segal and regularized Fredholm determinants and their relations are studied. See Quillen [27], Kontsevich & Vishik [30] and Scott & Wojciechowski [31] for more details.

Finally, in the study of the linear stability of travelling wave solutions to nonlinear partial differential equations often numerical simulation is required in the search for eigenvalues. Indeed, one of the main motivations for establishing the connection between the Evans function and Fredholm determinant was the need to compute the stability of multidimensional travelling waves (e.g. [32]). Gesztesy *et al.* [16, Section 4] established important convergence results for suitable Galerkin approximations for the multidimensional problem. Humpherys & Zumbrun [33], Ledoux *et al.* [34] and Ledoux *et al.* [35] have also designed numerical methods for computing the Evans function for the multidimensional case. The computation of Fredholm determinants also naturally arise in the computation of the Maslov index for linear symplectic systems, in particular in the multi-dimensional case which relies on computing flows in the Fredholm Lagrangian Grassmannian manifold [36,37]. Finally, a natural question to ask that is still open is, as numerical tools, which of the Evans function, transmission coefficient, Fredholm determinant or Galerkin-type direct projection methods has the optimal complexity? See Karambal [38] for some results in this direction. A comprehensive study of this complexity issue would be a useful follow-on project.

## Appendix A. Proof of the Hilbert–Schmidt class lemma

Proof. First, given $K\in I_{\infty }$, the existence of a kernel function $G\in L^{2}(R^{2};C^{n\times n})$ if and only if $K\in I_{2}$, follows from standard existence theory [17, p. 210]. Second, we prove the isometry of the map *G*↦*K* from $L^{2}(R^{2};C^{n\times n})$ to $I_{2}$. Suppose the set of $C^{n}$-valued functions {*φ*_*m*_}_*m*≥1_ are an unitary basis for $L^{2}(R;C^{n})$. The set ${\left\{\varphi_{\ell }⊗\varphi_{m}^{†}\right\}}_{\ell ,m\geq 1}$ is a unitary basis for the Hilbert space $L^{2}(R^{2};C^{n\times n})$. Hence for any $G\in L^{2}(R^{2};C^{n\times n})$, there exists a double sequence of constants $G_{\ell ,m}\in C^{n\times n}$ whose trace is square-summable, such that $G(x;y)=\sum_{\ell ,m\geq 1}G_{\ell ,m}\varphi_{\ell }(x)\varphi_{m}^{†}(y)$. By direct calculation, we have

$$\begin{array}{rl} \mathrm{tr}\;|K{|}^{2} & =\sum_{m\geq 1}{\left\langle \varphi_{m},K^{†}K\varphi_{m}\right\rangle }_{L^{2}(R;C^{n})}\\ & =\sum_{m\geq 1}{\left\langle K\varphi_{m},K\varphi_{m}\right\rangle }_{L^{2}(R;C^{n})}\\ & =\sum_{m\geq 1}\int_{R}{\left(K\varphi_{m}\right)}^{†}(x)(K\varphi_{m})(x)\;dx\\ & =\sum_{m\geq 1}\int_{R^{3}}(G(x,\xi )\varphi_{m}{\left(\xi )\right)}^{†}(G(x,\eta )\varphi_{m}(\eta ))\;d\xi \;d\eta \;dx\\ & =\sum_{m\geq 1}\int_{R^{3}}\varphi_{m}^{†}(\xi )G^{†}(x,\xi )G(x,\eta )\varphi_{m}(\eta )\;d\xi \;d\eta \;dx. \end{array}$$

Using the expansion for *G*=*G*(*x*;*y*) above we find,

$$\begin{array}{rl} \mathrm{tr}\;|K{|}^{2} & =\sum_{m,k,\ell ,p,q\geq 1}\int_{R^{3}}\varphi_{m}^{†}(\xi ){\left(G_{k,\ell }\varphi_{k}(x)\varphi_{\ell }^{†}(\xi )\right)}^{†}(G_{p,q}\varphi_{p}(x)\varphi_{q}^{†}(\eta ))\varphi_{m}(\eta )\;d\xi \;d\eta \;dx\\ & =\sum_{m,k,\ell ,p,q\geq 1}\int_{R^{3}}\varphi_{m}^{†}(\xi )\varphi_{\ell }(\xi )\varphi_{k}^{†}(x)G_{k,\ell }^{†}G_{p,q}\varphi_{p}(x)\varphi_{q}^{†}(\eta )\varphi_{m}(\eta )\;d\xi \;d\eta \;dx\\ & =\sum_{m,k,p\geq 1}\int_{R}\varphi_{k}^{†}(x)G_{k,m}^{†}G_{p,m}\varphi_{p}(x)\;dx\\ & =\mathrm{tr}\;\sum_{m,k,p,r\geq 1}\int_{R^{2}}\varphi_{m}(y)\varphi_{k}^{†}(x)G_{k,m}^{†}G_{p,r}\varphi_{p}(x)\varphi_{r}^{†}(y)\;dx\;dy\\ & =\mathrm{tr}\;\sum_{m,k,p,r\geq 1}\int_{R^{2}}{\left(G_{k,m}\varphi_{k}(x)\varphi_{m}^{†}(y)\right)}^{†}(G_{p,r}\varphi_{p}(x)\varphi_{r}^{†}(y))\;dx\;dy\\ & =\int_{R^{2}}\mathrm{tr}\;G^{†}(x;y)G(x;y)\;dx\;dy\\ & =∥G{∥}_{L^{2}(R^{2};C^{n\times n})}^{2}. \end{array}$$

▪

## Appendix B. Proof of the trace formula lemma

Proof. For any ℓ∈N, suppose that *K*_1_,*K*_2_,…,*K*_ℓ_ are Hilbert–Schmidt operators with canonical respective kernels *G*_1_,*G*_2_,…,*G*_ℓ_. As in the proof of the Hilbert–Schmidt class lemma above, there exists a double sequence of constants $G_{p,m}^{\left(\ell \right)}\in C^{n\times n}$ whose trace is square-summable, such that $G_{\ell }(x;y)=\sum_{p,m\geq 1}G_{p,m}^{\left(\ell \right)}\varphi_{p}(x)\varphi_{m}^{†}(y)$. Then by direct computation, we see that

$$\begin{array}{rl} \mathrm{tr}\;K_{1}\cdots K_{\ell } & =\sum_{m\geq 1}{\left\langle \varphi_{m},K_{1}\cdots K_{\ell }\varphi_{m}\right\rangle }_{L^{2}(R;C^{n})}\\ & =\sum_{m\geq 1}\int_{R}\varphi_{m}^{†}(x)(K_{1}\cdots K_{\ell }\varphi_{m})(x)\;dx\\ & =\sum_{m\geq 1}\int_{R^{\ell +1}}\varphi_{m}^{†}(x)G_{1}(x;y_{1})G_{2}(y_{1};y_{2})\cdots G_{\ell }(y_{\ell -1};y_{\ell })\varphi_{m}(y_{\ell })\;dy_{\ell }\cdots dy_{1}\;dx\\ & =\sum_{m,p,q\geq 1}\int_{R^{\ell +1}}\varphi_{m}^{†}(x)G_{1}(x;y_{1})\cdots \\ & \;\cdots G_{\ell -1}(y_{\ell -2};y_{\ell -1})G_{p,q}^{\left(\ell \right)}\varphi_{p}(y_{\ell -1})\varphi_{q}^{†}(y_{\ell })\varphi_{m}(y_{\ell })\;dy_{\ell }\cdots dy_{1}\;dx\\ & =\sum_{m,p\geq 1}\int_{R^{\ell }}\varphi_{m}^{†}(x)G_{1}(x;y_{1})\cdots G_{\ell -1}(y_{\ell -2};y_{\ell -1})G_{p,m}^{\left(\ell \right)}\varphi_{p}(y_{\ell -1})\;dy_{\ell -1}\cdots dy_{1}\;dx\\ & =\mathrm{tr}\;\int_{R^{\ell }}\sum_{m,p\geq 1}G_{1}(x;y_{1})\cdots G_{\ell -1}(y_{\ell -2};y_{\ell -1})G_{p,m}^{\left(\ell \right)}\varphi_{p}(y_{\ell -1})\varphi_{m}^{†}(x)\;dy_{\ell -1}\cdots dy_{1}\;dx\\ & =\mathrm{tr}\;\int_{R^{\ell }}G_{1}(x;y_{1})G_{2}(y_{1};y_{2})\cdots G_{\ell -1}(y_{\ell -2};y_{\ell -1})G_{\ell }(y_{\ell -1};x)\;dy_{\ell -1}\cdots dy_{1}\;dx\\ & =\mathrm{tr}\;\int_{R^{\ell }}G_{1}(y_{1};y_{2})G_{2}(y_{2};y_{3})\cdots G_{\ell -1}(y_{\ell -1};y_{\ell })G_{\ell }(y_{\ell };y_{1})\;dy_{\ell }\cdots dy_{1}. \end{array}$$

The proof for the case of a single trace class operator *K* with continuous kernel *G* exactly follows the argument above with ℓ=1.▪
